# Engineering Thermoresponsive In Situ Gels Incorporating Nutraceutical-Laden Nanostructured Lipid Carriers for Controlled Periodontal Drug Release

**DOI:** 10.3390/gels12040268

**Published:** 2026-03-24

**Authors:** Rabia Ashfaq, Anita Kovács, Szilvia Berkó, Gábor Katona, Rita Ambrus, Tamás Ferenc Polgár, Mária Szécsényi, Katalin Burián, Mária Budai-Szűcs

**Affiliations:** 1 Institute of Pharmaceutical Technology and Regulatory Affairs, Faculty of Pharmacy, University of Szeged, H-6720 Szeged, Hungarygasparne.kovacs.anita@szte.hu (A.K.); berko.szilvia@szte.hu (S.B.); katona.gabor@szte.hu (G.K.); ambrus.rita@szte.hu (R.A.); 2 Core Facility, HUN-REN Biological Research Centre, H-6726 Szeged, Hungary; polgar.tamas@brc.hu; 3 HUN-REN-SZTE Neuroscience Research Group, Hungarian Research Network, University of Szeged, H-6725 Szeged, Hungary; 4 Department of Medical Microbiology, University of Szeged, H-6720 Szeged, Hungary; szecsenyi.maria@med.u-szeged.hu (M.S.); burian.katalin@med.u-szeged.hu (K.B.)

**Keywords:** periodontitis, clove essential oil, apigenin, NLC, in situ gel, poloxamer, Raman mapping, SEM

## Abstract

Periodontitis is a chronic inflammatory disease marked by the progressive destruction of periodontal tissues, where conventional therapies often fail to maintain adequate drug levels at the target site. This study reports the development and characterization of a thermosensitive gel containing nanostructured lipid carriers (NLC) for controlled local periodontal delivery. Apigenin (AP)-loaded NLC were prepared using AP as active agent and clove essential oil (CEO) as liquid lipid subsequently incorporated into Poloxamer 407 (5–15% *w*/*w*) hydrogels. The formulations were evaluated in relation to particle size, morphology, thermal and rheological behavior, mucoadhesion, in vitro release, antibacterial activity, and stability. Optimized nanoscale NLC showed a high entrapment efficiency, and uniform morphology. Raman analysis confirmed successful AP incorporation and homogeneous distribution in the gel without incompatibility. NLC-loaded gels exhibited sol–gel transition at physiological temperature with improved viscoelasticity and enhanced mucoadhesion. The drug release was sustained for 48 h and followed the Korsmeyer–Peppas model, indicating diffusion-based and anomalous transport. The antibacterial assessment demonstrated the pronounced inhibitory activity of the NLC formulations against key periodontal pathogens, with the formulation-dependent modulation of antimicrobial efficacy observed following the gel incorporation. Stability studies showed preserved nanoparticle structure and uniform dispersion. Overall, the thermoresponsive NLC-hydrogel system offers a promising strategy for prolonged, localized periodontal therapy.

## 1. Introduction

Periodontitis is a common chronic inflammatory disease that affects the supporting structures of the teeth, including the gingiva, periodontal ligament, and alveolar bone. It is primarily driven by bacterial infection and an exaggerated host immune response, which, if not properly managed, can lead to progressive tissue destruction and eventual tooth loss [1,2]. Conventional periodontal therapies, including dental biofilm control, mechanical debridement, and the administration of local or systemic antibiotics, often demonstrate limited clinical effectiveness due to factors such as inadequate drug penetration into periodontal pockets and the occurrence of systemic side effects. Amoxicillin, tetracycline, doxycycline, minocycline, clindamycin, or amoxicillin combined with metronidazole is commonly prescribed as the antibiotic to manage advanced periodontitis [3]. However, the increasing prevalence of antibiotic resistance frequently undermines its therapeutic efficacy, necessitating the exploration of novel therapeutic approaches [4].

In recent years, nanotechnology-based drug delivery systems have emerged as a promising strategy to overcome these challenges. Among them, nanostructured lipid carriers (NLC) have gained significant attention due to their unique composition of both solid and liquid lipids, which enhances drugs’ loading, stability, and controlled release. NLC can be engineered to improve drug bioavailability, provide sustained therapeutic action, and target inflamed periodontal tissues more effectively [5]. Collectively, these characteristics establish NLC as highly effective delivery platforms capable of enhancing periodontal treatment strategies and contributing to improved therapeutic outcomes for patients.

In the present study, clove essential oil (CEO) was selected as the medicated liquid lipid phase due to its multiple therapeutic properties that are highly relevant for periodontal treatment. Derived from the flower buds of *Syzygium aromaticum*, CEO is rich in eugenol (EUG), a phenylpropanoid with strong antimicrobial, anti-inflammatory, and antinociceptive activities [6]. EUG has been shown to inhibit the growth of various periodontopathogens, reduce local inflammation by modulating cytokine release, and provide pain relief at the site of infection [7]. At higher doses, EUG has been found to exhibit anesthetic properties [8]. Due to its antioxidant, anti-inflammatory, and pain-relieving effects, EUG is widely utilized in dental applications at lower concentrations. Incorporating CEO in lipid-based formulations enhances the dissolution profile of lipophilic drugs, thereby increasing absorption and preventing oxidative degradation [9].

As an active agent, apigenin (AP) was incorporated into NLC (NLC-AP) to augment the therapeutic efficacy. AP is a naturally occurring flavonoid found in many fruits and vegetables, particularly parsley, chamomile, and celery [10]. It possesses a wide spectrum of pharmacological properties, including antioxidant, anti-inflammatory, antibacterial, and antiproliferative effects [11,12]. AP exerts anti-inflammatory effects by downregulating key pro-inflammatory mediators, including nitric oxide (NO), inducible nitric oxide synthase (iNOS), prostaglandin E_2_ (PGE_2_), cyclooxygenase-2 (COX-2), and tumor necrosis factor-α (TNF-α), primarily through the inhibition of the nuclear factor-κB (NF-κB) and signal transducer and activator of transcription (STAT) signaling pathways [13,14]. Its combined use with CEO in a nanostructured lipid-based system provides a synergistic approach to the management of periodontitis by targeting both the microbial and inflammatory components of the disease, as has already been established by our research group [15].

Together, the integration of CEO and AP into a nanocarrier system represents a novel and multifaceted strategy to improve local drug delivery, sustain therapeutic action, and improve clinical outcomes in the treatment of periodontitis. However, to further prolong their retention at the disease site and ensure sustained drug release, NLC can be incorporated into hydrogels.

In certain cases, the hydrogel is initially administered in liquid form and subsequently transforms into a gel once inside the body, due to its ability to undergo sol–gel transitions in response to specific biological triggers [16]. This characteristic offers several benefits, including their simple preparation due to their fluid nature at room temperature, gel formation at the site of administration, extended retention in the target area, and the controlled and sustained release of therapeutic agents [17,18]. For this reason, pharmaceutical research has increasingly concentrated in recent decades on creating stimuli-responsive hydrogels for a wide range of applications [19].

Poloxamers, generally recognized as safe (GRAS) and commercially designated as Pluronics^®^ or Lutrol^®^, represent a category of synthetic polymers characterized by their triblock architecture. These amphiphilic copolymers exhibit non-ionic, water-soluble properties and adopt alternating polymer sequences. Within these structures, “poly(ethylene oxide) (PEO)” blocks are hydrophilic polymers and “poly(propylene oxide) (PPO)” are their hydrophobic counterpart [20]. This distinct arrangement confers dual functionality: the PEO segments impart solubility in aqueous environments, whereas the PPO domains drive hydrophobic interactions. This molecular design underpins their versatility in applications requiring controlled self-assembly or temperature-responsive behavior.

Recent research has focused extensively on exploring the drug delivery potential of Poloxamer 407 (P407) in the management of periodontal disease. P407 is an FDA-approved excipient commonly used in a range of pharmaceutical formulations [21]. This has led to the development of multiple in situ gelling systems designed for localized drug delivery to periodontal tissues. These formulations leverage the thermoresponsive behavior of P407 to form gels at physiological temperatures, enabling the sustained release of antimicrobial or anti-inflammatory agents directly at the disease site. Such advances aim to enhance treatment efficacy by enhancing drug retention and reducing systemic side effects [22,23]. Thermoresponsive hydrogels based on P407 have also been extensively studied for mucosal drug delivery, due to their gentle interaction with biological membranes and their ability to target specific sites, maintain therapeutic drug levels over time, and potentially reduce both the effective dose and associated side effects [24]. These molecules can interact with mucus by forming physical entanglements or non-covalent interactions, which enhances their adhesion to biological tissues and helps extend the duration of the formulation at the site of application [25].

The concentration of P407 plays a critical role in determining the sol–gel transition behavior and rheological properties of thermoresponsive systems. Based on literature reports and a preliminary formulation screening, a concentration range of 5–15% (*w*/*w*) was selected for this study. Lower polymer levels were explored because NLC has been shown to enhance the rheological behavior of gelling systems [26] and to potentially enable gel formation even at reduced polymer concentrations.

The aim of this study is to develop a thermosensitive, in situ gelling system based on P407 that incorporates optimized AP-loaded NLC in which CEO is used as bifunctional liquid lipid for localized periodontal delivery [15]. The formulation was designed to remain liquid at room temperature and undergo gelation at physiological temperature to enhance the retention at the site of application. The system was comprehensively characterized with respect to the physicochemical and morphological properties of the NLC, followed by a comparative evaluation of blank and NLC-loaded gels in terms of the gelation temperature, gelation time, storage modulus, loss factor, and mucoadhesive properties. Furthermore, the in vitro drug release of AP and the antibacterial activity of CEO and AP were assessed, and stability studies were conducted to assess the formulation integrity, sustained drug release behavior, and suitability for prolonged local therapy.

## 2. Results and Discussion

### 2.1. Nanoparticle Physical Characterization

The previously optimized AP-loaded NLC formulations comprising AP and CEO [15] were characterized to assess their physicochemical performance (Table 1). The NLC-AP formulation exhibited a mean hydrodynamic diameter (PS) of 188.5 nm and a narrow particle size distribution (PDI), demonstrating good homogeneity. The zeta potential (ZP) of NLC-AP was −17 mV, indicating the moderate electrostatic stabilization of the colloidal dispersion. Furthermore, the high values of EE and DL confirmed the strong affinity of AP for the lipid matrix. This enhanced encapsulation can be attributed to the lipophilic nature of AP, which favors its incorporation into lipid-rich systems and supports increased drug-loading capacity.

For the formulations NP-5, NP-7, NP-10, and NP-15, EE was consistently high, ranging from 96% to 98% (Table 2), while DL values were between 4.7% and 4.9%. When NLC-AP was incorporated into the gel, the %EE appeared higher than that of the standalone NLC-AP system. This elevated EE can be attributed to the absence of a purification step prior to gel loading. The NLC dispersion was directly incorporated into the polymeric gel matrix without removing any unencapsulated (free) drug. As a result, drug molecules present outside the nanoparticles were retained within the gel, leading to an overestimation of the apparent EE. This also explains the expected initial burst release, since the free drug embedded in the gel matrix is readily available for rapid release.

### 2.2. TEM Analysis of Nanoparticles

**Figure 1:**
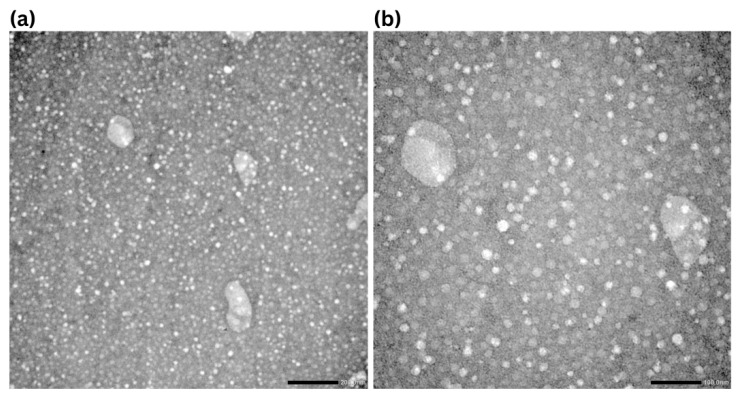
Representative TEM micrographs at magnifications of 25,000× (**a**) and 50,000× (**b**) with corresponding scale bars of 200 and 100 nm, respectively.

The morphology and size distribution of the NLC-AP was examined using TEM. Micrographs at 25,000× (Figure 1a) and 50,000× (Figure 1b) revealed roughly spherical or ellipsoidal nanoparticles with smooth surfaces. The particles appeared to be relatively uniform and well dispersed, with no significant aggregation observed across the visual field. At higher magnifications, the images showed a population of discrete nanoparticles in the nanometer range, along with occasional larger structures. These observations were consistent with nanoscale dimensions, as indicated by the 200 nm and 100 nm scale bars.

The nanoparticles exhibited an average size distribution reaching up to 160 nm, consistent with the expected nanoscale range and closely matching the values obtained from DLS analysis. The observed discrepancy in particle size arises from the different measurement techniques. TEM captures the diameter of particles in the dry state, whereas DLS determines the average size of particles in their hydrated form, including the solvation layer [27].

### 2.3. DSC of Nanoparticles and Loaded Gels

**Figure 2:**
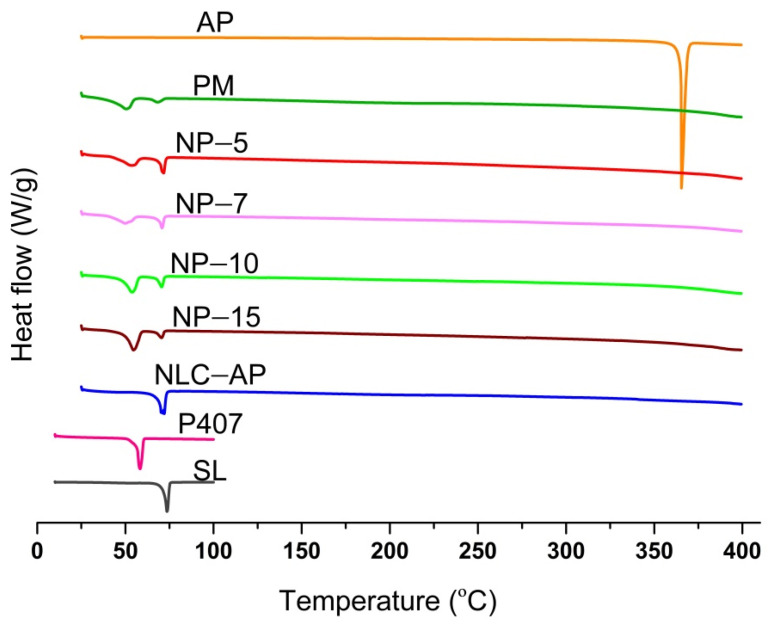
Thermograms of the formulations, the physical mixture, and the raw materials.

The thermal behavior of the individual components, the physical mixture (PM), and various formulations of NLC-AP was evaluated using DSC, and the resulting thermograms are presented in Figure 2. AP displayed a sharp endothermic peak at approximately 365 °C [28], corresponding to its melting point and indicative of its crystalline nature. According to the literature, AP exhibits a sharp endothermic peak at approximately 366 °C, corresponding to its melting point, while TGA analysis indicates that thermal decomposition begins within a similar temperature range (~347–600 °C) [29]. Similarly, another study mentioned that the DSC thermogram of AP showed a sharp crystalline peak at a fusion temperature of 366.4 °C, while TGA analysis revealed a minor mass loss of 1.872% (*w*/*w*) around 342 °C, attributed to the onset of thermal decomposition [30]. Together, these findings indicate that while the DSC peak reflects the melting transition of AP, initial decomposition may occur concurrently at this temperature. SL exhibited a broad melting peak around 73.04 °C, in close agreement with the previously reported data [31], while P407 showed a distinct endothermic peak at 58.34 °C, between 55 and 60 °C, as published in the literature [32], confirming their individual characteristic thermal profiles. The thermogram of NLC-AP shows a broad, reduced-intensity endothermic event at 71.86 °C. This observed decrease in the melting temperature of SL in both the PM and nanoformulations probably reflects hydrophobic interactions between the lipid components. The mixing of the liquid and solid lipids can alter the crystal structure of the solid lipid, thus causing delayed recrystallisation and improving the physical stability [33]. The absence of any distinct phase transitions from the individual components further confirms the successful formation of stable NLC. In the NLC-loaded gel formulations, increasing concentrations of P407 led to a gradual reduction in the melting point of SL. In case of nanogels (NP-5, NP-7, NP-10, and NP-15) the endothermic peaks of SL were shifted to lower temperatures of 71.63 °C, 70.79 °C, 70.68 °C, and 70.52 °C, respectively. The extent of the suppression of the SL peak increased with higher P407 concentrations, suggesting that P407 reduces the crystallinity of the lipid matrix, likely due to effective lipid interactions enabled by the hydrophobic PPO central block of the poloxamer [34].

In particular, the characteristic melting peak of AP disappeared across all NLC formulations. DSC analysis indicated that this disappearance was due to the transformation of AP from a crystalline state to an amorphous or molecularly dispersed state within the lipid matrix.

### 2.4. Raman Spectroscopy

Raman spectroscopy was used to investigate the molecular interactions, structural integrity and compatibility of AP within the NLC system and its incorporation into P407-based hydrogels. The Raman spectrum of AP exhibited distinct and sharp characteristic bands at ~1200–1300 cm^−1^ (C–O stretching and phenolic vibrations) and ~1500–1650 cm^−1^ (aromatic C=C stretching of the flavonoid ring). Similarly to other flavone derivatives, the intense band near 1600 cm^−1^ corresponds to the C=O stretch, coupled with C2=C3 or quinoidal ring stretching vibrations [35]. These intense, narrow peaks confirm the crystalline nature of pure AP.

In the Raman spectrum of CEO, a characteristic signal is observed near 1639 cm^−1^, corresponding to the stretching vibration of the C=C bond. In addition, an intense peak that appears at approximately 1293 cm^−1^ is associated with the rocking vibration of the =CH group. EUG, which possesses an ether functional moiety, typically exhibits distinct Raman features related to aromatic ether vibrations. These include bands attributed to Ar–O (where Ar denotes a benzene ring) stretching modes within the range of 1300–1210 cm^−1^, as well as C–O stretching vibrations appearing between 1050 and 1010 cm^−1^. Consequently, the peaks detected at 1273 cm^−1^ and 1036 cm^−1^ are assigned to the Ar–O and C–O stretching vibrations of EUG, respectively. Moreover, a band observed around 900 cm^−1^ is characteristic of the wagging motion of the monoalkyl ethylene group (R–CH=CH_2_) in the Raman spectrum [36]. The relatively broad peaks indicate the amorphous and liquid nature of CEO.

The Raman spectrum of SL showed typical vibrations related to lipids at ~1060–1130 cm^−1^ (C–C stretching of alkyl chains), ~1290–1300 cm^−1^ (CH_2_ twisting), and ~1440 cm^−1^ (CH_2_ scissoring) [37]. These peaks confirm the presence of a solid lipid matrix. The Raman spectrum of NLC-AP showed noticeable changes compared to pure AP, with a reduced intensity of the characteristic peaks of AP. An overall broadening of the peak and slight shifts in the region of 1200–1650 cm^−1^ were observed, along with the overlap of the AP peaks with lipid-associated bands. These observations indicate that AP is molecularly dispersed within the lipid matrix, and that weak interaction forms such as hydrophobic or H-bonds. The absence of new peaks suggests no strong chemical interaction or incompatibility between the NLC components. All hydrogel formulations showed a further suppression and broadening of the AP peaks, indicating enhanced encapsulation and reduced crystallinity. With the increasing concentration of P407, the characteristic AP bands became progressively less prominent, particularly in NP-10 and NP-15. The Raman profiles were dominated by broad bands, reflecting the semisolid and amorphous nature of the hydrogel network. No new Raman bands were observed across the formulations, confirming the compatibility between AP, NLC and P407. The gradual decrease in the AP peak intensity with the increasing concentration of P407 suggests improved drug entrapment and a homogeneous distribution of NLC-AP within the hydrogel matrix.

**Figure 3:**
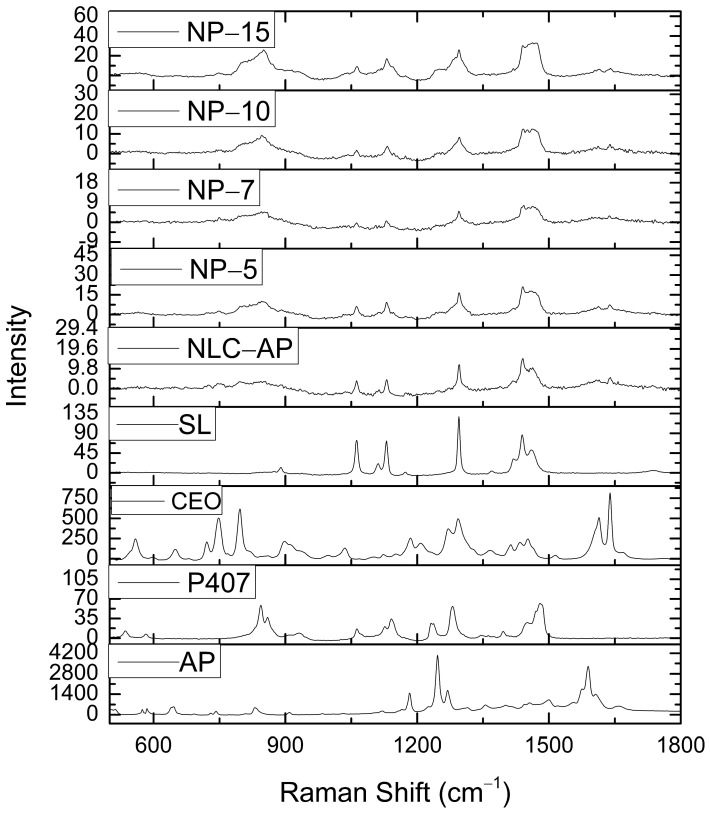
Raman spectrum peaks of raw materials, nanoparticles, and nanoparticle-embedded thermosensitive gels.

In conclusion, Raman spectroscopy confirmed the successful incorporation of AP in the NLC and its uniform distribution within P407-based hydrogels. In Figure 3, the characteristic Raman bands of AP in the 1200–1650 cm^−1^ region showed reduced intensity, peak broadening, and slight shift in NLC-AP- and NLC-AP-loaded hydrogels (NP-5, NP-7, NP-10, and NP-15), indicating the loss of crystallinity and possible molecular dispersion of the drug within the lipid–hydrogel matrix. No additional peaks were detected, indicating the absence of chemical interactions and confirming that AP is physically incorporated within a stable NLC-hydrogel system.

Raman mapping allows for the visualization of the spatial distribution of the chemical components within a system. A hyperspectral map consists of Raman spectra recorded at discrete locations, with each spectrum corresponding to a specific position on the sample’s surface [38]. Raman mapping was performed to evaluate the spatial distribution and physicochemical state of active compounds (AP and CEO) and lipids through the critical stages of an advanced formulation process: (i) the formation of CEO-loaded NLC (NLC–CO), (ii) the encapsulation of AP within NLC–CO (NLC-AP), and (iii) the subsequent incorporation of these NLC-AP into the P407 hydrogel matrix. The results confirm successful nanoparticle encapsulation, uniform component distribution, and a preserved microstructure within the formulation, with the normalized intensity bar highlighting compositional the homogeneity.

**Figure 4:**
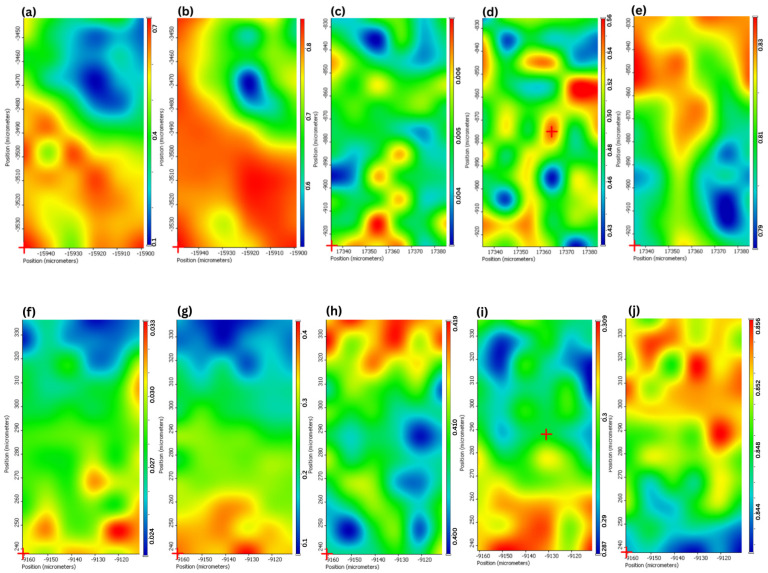
Raman mapping for the NLC–CO components: (**a**) CEO and (**b**) SL; NLC-AP components: (**c**) AP, (**d**) CEO and (**e**) SL; and NP-10 components: (**f**) AP, (**g**) CEO, (**h**) SL, (**i**) NLC-AP, and (**j**) P407. The scale bar represents the normalized intensity in arbitrary units (a.u.).

The initial Raman map of NLC–CO served as a foundational control. The distribution of the characteristic C=C aromatic stretch of EUG was found to be uniformly dispersed throughout the SL matrix. The normalized intensity showed a narrow distribution, predominantly in the midrange values, confirming the successful formation of NLC where the liquid lipid (CEO) is effectively integrated into the solid lipid matrix without phase separation Figure 4a,b. This homogeneity is the cornerstone of efficient drug loading in subsequent steps, ensuring a consistent and predictable environment for the active ingredient. A microscopic image of the lyophilized sample, illustrating the surface analyzed by Raman mapping, is provided in the Supplementary File (Figure S1).

The spatial distributions for both AP (via a unique carbonyl/aromatic ring vibration) and CEO were independently homogeneous, confirming that the addition of the flavonoid did not disrupt the integrity of the NLC matrix. The maps for AP and CEO showed a high degree of spatial correlation (Figure 4c,d). This may show that AP is not randomly dispersed within the solid lipid but is preferentially dissolved or molecularly dispersed within the liquid lipid domains of the NLC. The incorporation of the NLC-AP into the P407 hydrogel represents the final step in the formulation. The Raman map successfully detected the distinct signals of AP, CEO, SL, and NLC-AP, confirming the presence of the NLC within the gel matrix (Figure 4f–i). The key finding is that the co-distribution of AP and CEO remained consistent, highlighting their stable association. Furthermore, the NLC-AP system is homogeneously dispersed within the P407 hydrogel, suggesting that the incorporation into the gel does not induce aggregation or structural disruption.

Overall, Raman mapping highlights the consistent co-distribution of AP and CEO and the homogeneous dispersion of the NLC-AP system within the P407 hydrogel, indicating preserved structural integrity without aggregation. These findings, demonstrating stable drug distribution and formulation integrity, are further supported by in vitro release, antibacterial, and morphological studies, confirming the robustness of the system.

### 2.5. Rheological Analysis

Initially, the sol–gel transition temperature of P407-containing formulations’ data revealed an inverse relationship between polymer concentration and temperature, where a higher P407 content resulted in a lower transition temperature. This phenomenon is attributed to the thermos-responsive micellization mechanism of P407. At lower temperatures, the polymer exists as individual unimers (monomers) in solution; the polymer chains are highly hydrated and remain dispersed in the aqueous medium. Upon heating, the hydrophobic poly(propylene oxide) (PPO) blocks undergo desolvation, an endothermic process that promotes hydrophobic interactions within the copolymer blocks, leading to the aggregation of unimers into spherical micelles. Once a critical micelle concentration and temperature are reached, these micelles pack into a three-dimensional network, triggering gelation [25,39]. Thus, the increased polymer concentration accelerates micelle formation and network assembly, lowering the temperature required for the sol-to-gel transition.

The gelling properties of the formulations are summarized in Table 3. The gelling temperature of the nanoparticle-loaded gels ranged from below 15 °C to approximately 33 °C, depending on the P407 concentration. Specifically, NP-5 gelled at approximately 29.84 °C, while NP-7 and NP-10 formulations gelled at around 30.64 °C and 27.24 °C, respectively. NP-15 exhibited rapid gelation at temperatures below 15 °C. In contrast, the blank gels (P) displayed gelling temperatures slightly higher than their counterparts loaded with nanoparticle at similar concentrations, with P-5 at 34.84 °C, P-7 at 33.04 °C, P-10 at 28.64 °C, and P-15 at 22.87 °C.

The gelling times decreased with increasing P407 concentrations and were further influenced by the presence of nanoparticles. NP-5 gelled in 1.4 min, whereas P-5 required almost 5.8 min. At 7%, NP-7 gelled in one minute, compared to 3.3 min for P-7. The 10% formulations showed similar trends, as NP-10 gelled within a minute while P-10 took 2.4 min. The 15% formulations exhibited robust gelation, with P-15 solidifying in approximately 1 min, while NP-15 was already in a gel state due to the incorporated NLC.

The gel strengths, assessed through storage modulus (G′) and loss factor (tan δ), were promising in nanoparticle-loaded systems at all concentrations. At 10 rad/s, corresponding to the reported chewing frequency [40], NP-5 exhibited a G′ of approximately 3703 Pa, compared to 1191 Pa for P-5. Similarly, NP-7 showed a G′ of about 4620 Pa versus 3486 Pa for P-7. NP-10 exhibited a G′ of approximately 4962 Pa, while this was 2424 Pa in the case of P-10. The 15% formulations showed the highest G′, with NP-15 reaching approximately 14,468 Pa, markedly higher than P-15 at around 7969 Pa. In general, increasing the P407 concentration and the incorporation of nanoparticles enhanced the gel strength and gelation speed. This transition is identified through the loss tangent (tan δ), where tan δ < 1 indicates elastic-dominant (solid-like) behavior, while tan δ > 1 represents viscous-dominant (liquid-like) behavior [41,42]. Therefore, the loss factor provides critical insight into the viscoelastic nature of the gel. Lower values of the loss factor signify a more elastic-dominant viscoelastic response, whereas higher values indicate increased viscous contribution. The Supplementary File contains all the temperature- and time-dependent sweep curves (Figure S2a–h).

Nanoparticles can interact with P407 micelles or polymer chains through physical or chemical interactions. In our DSC and Raman spectrum analysis we could observe some changes that can correspond to some weak interactions (like hydrophobic and or H-bonds). These interactions may stabilize the gel network, requiring a physiological temperature to trigger gelation. The presence of nanoparticles can influence the self-assembly process of P407 molecules, potentially disrupting micelle formation or modifying the micelle packing density. Overall, nanoparticles can modify the local environment around P407 molecules, altering the hydrophobic–hydrophilic balance. This can affect the temperature at which micelles aggregate into a gel network. Based on the above results, it can be concluded that the NP-10 formulation is the most suitable for gelation at body temperature, exhibiting optimal gel strength, favorable viscoelastic properties, and a rapid gelation time.

### 2.6. Mucoadhesive Study

Mucoadhesion refers to the prolonged adhesion between two surfaces, where at least one is biological, such as the interaction between a dosage form and the mucosal surface. This occurs through the wetting and adsorption of hydrogels, allowing their polymer chains to diffuse into and interpenetrate the mucosa. These chains form supramolecular interactions such as hydrogen bonds, electrostatic forces, or hydrophobic contacts with mucosa [43].

**Figure 5:**
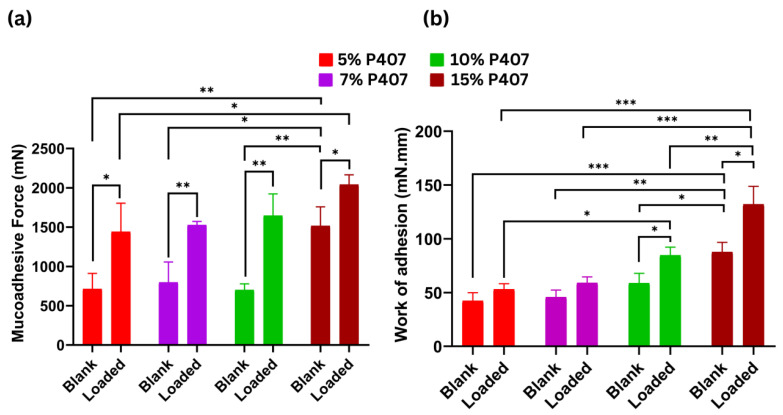
Comparison of the (**a**) mucoadhesive force and (**b**) work of adhesion of blank and NLC-loaded gels formulated with different concentrations of P407 (5%, 7%, 10%, and 15%). Data are expressed as the mean ± SD. The statistical differences between blank and corresponding NLC-loaded gels were analyzed using Student’s *t* test. Comparisons among formulations with different concentrations of P407 were performed using one-way ANOVA followed by Tukey’s multiple comparison test. Levels of statistical significance are indicated as *p* < 0.05 (*), *p* < 0.01 (**), and *p* < 0.001 (***).

Figure 5a likely depicts a force–displacement curve measuring the adhesive strength of P407. The results indicate a high peak mucoadhesive force, suggesting a strong interaction between the polymer and the mucosal surface. This force is critical for ensuring prolonged adhesion in dynamic physiological environments. Figure 5b represents the work of adhesion (the area under the force curve) as a demonstration of P407 adhering to mucosal tissue. The data highlight a significant work of adhesion, reflecting the energy required to detach the polymer from the mucosa. This parameter correlates with the formulation’s ability to maintain sustained contact, a key factor for extended drug delivery.

Mucoadhesive properties increased in a concentration-dependent manner for both blank (P) and nanoloaded (NP) poloxamer gels, with NP formulations consistently exhibiting a superior performance. At the lowest concentration (5%), NP-5 showed a comparatively lower mucoadhesive work (53 mN·mm) and force (1442.6 mN), while the corresponding blank gel (P-5) demonstrated minimal mucoadhesion (42.3 mN·mm and 71.3 mN). At an intermediate concentration, NP-10 displayed markedly enhanced mucoadhesive behavior, with a work of 84.6 mN·mm and a force of 1646 mN, indicating strong gel–mucus interactions. The highest mucoadhesive performance was observed at a concentration of 15% poloxamer, with NP-15 showing a mucoadhesive work of 132 mN·mm and a force of 2041 mN, significantly exceeding the corresponding blank gel (P-15: 87.6 mN·mm and 1517 mN). Overall, these results confirm a clear concentration-dependent increase in mucoadhesion, further amplified by nanoparticle incorporation.

The observed mucoadhesive behavior of P407 can be attributed to its thermoreversible gelation behavior and physical interaction with mucosal glycoproteins. P407, a triblock copolymer (PEO-PPO-PEO), undergoes sol–gel transitions at physiological temperatures, enhancing its entrapment within mucosal crevices. This facilitates hydrogen bonding and chain entanglement, both critical for robust mucoadhesion, as is mentioned in the literature, where hyaluronic acid interacts through OH and –COOH groups and flexible chains interweaving within the mucin network [44]. The work of adhesion further underscores its potential for prolonged drug release, as higher energy dissipation during detachment implies an extended residence time at the target site (e.g., nasal, buccal, or ocular mucosa). Based on rheological tests, the nanolipid-P407 combination strengthened the gel structure, which also had a positive effect on the mucoadhesive properties. These findings highlight the P407-nanoparticle combination as a promising candidate for mucoadhesive drug delivery systems, particularly for localized therapies requiring sustained release and reduced dosing frequency. Further investigations of biocompatibility and in situ gel stability under physiological conditions are warranted.

### 2.7. In Vitro Release Study

**Figure 6:**
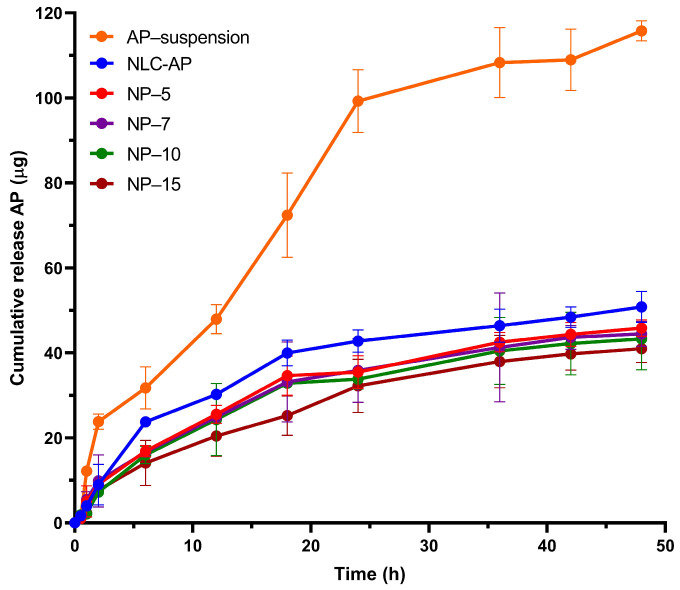
Release of AP from nanoparticles and gels loaded with nanoparticles in comparison to the suspension of AP constituted with P407 as the suspending agent.

The cumulative release profiles of NLC-AP, AP suspension prepared with 15% P407, and various NLC gel formulations over 48 h are presented in Figure 6. Cumulative release was expressed in micrograms (µg) from each formulation obtained at multiple time points. NLC-AP exhibited a sustained release profile, with a cumulative release of approximately 50.83 µg after 48 h. This slow and continuous release may be attributed to the encapsulation of AP in the lipid matrix, which is designed to provide a prolonged therapeutic effect. The initial release rate was slow, but gradually increased, reaching a plateau around 36 h. This sustained release profile is ideal for applications where a long-lasting therapeutic effect is desired, such as in chronic conditions. On the contrary, the AP suspension demonstrated the fastest release profile, exhibiting an initial release of ~12.17 µg at 1 h and reaching ~115.77 µg at 48 h, indicating rapid diffusion in the absence of nanoparticles. The suspension achieved a significant release (more than 50%) within the first 18 h. This rapid release may be due to the sink condition, the diffusion of the release medium into the dialysis bag, and/or the solubilizing effect of P407, which may enhance the dissolution rate of hydrophobic drugs in the aqueous medium [45]. However, such a fast release may limit the duration of action, making it suitable for conditions where rapid onset is more important than prolonged release.

The incorporation of NLC-AP in P407 gels resulted in a sustained and concentration-dependent release pattern. At 48 h, the cumulative release of AP from NLC-AP-loaded gels was approximately 45.85 µg, 44.48 µg, 43.29 µg, and 40.98 µg for P407 gels, respectively. Increasing the P407 concentration led to a gradual reduction in the release rate, which can be attributed to an increase in the gel viscosity and a denser cross-linked 3D polymeric structure that restricted the diffusion of NLC and drug molecules [46]. As mentioned in the literature, higher polymer levels lead to a denser gel network, which acts as a stronger physical barrier and slows down the diffusion of the drug. This behavior is further explained by the rise in viscosity at increased polymer concentrations [47]. Conclusively, the NLC-AP-loaded polymeric gels demonstrated a controlled and sustained release profile suitable for prolonged drug delivery. Although the in vitro release study was conducted for 48 h, the observed diffusion-controlled release behavior, together with the gel’s mucoadhesive and structural characteristics, suggests the potential to extend drug release beyond this period, potentially up to 4–5 days. This prolonged release is expected to support continuous antimicrobial activity within the periodontal pocket, thereby enhancing bacterial control and reducing the dosing frequency, which may ultimately improve patient compliance.

To further understand the release mechanisms, the release data were fitted to several kinetic models: Zero-order, First-order, Higuchi, and Korsmeyer–Peppas models. The best-fitting model for each formulation was evaluated on the basis of R-square values (Table 4). In the Korsmeyer–Peppas model, fractional drug release (Mt/M∞) is described as a power law function of time (t), where the release exponent (n) characterizes the dominant drug transport mechanism within polymeric matrices. The constant K serves as a kinetic parameter that encapsulates the structural and geometric attributes of the drug delivery system, and its units are dependent on the value of n, reflecting the time dependency of the release process [48].

An *n* value between 0.45 and 0.89 signifies anomalous (non-Fickian) release behavior, in which diffusion is the dominant mechanism. In this regime, matrix swelling reaches equilibrium more rapidly than drug liberation, resulting in diffusion occurring primarily through the pre-swollen polymer network [49]. The release from NLC-AP followed the Korsmeyer–Peppas model, indicative of Fickian diffusion. The model provided a good fit to the release data (R^2^ = 0.97), suggesting that drug release is primarily governed by diffusion through the lipid matrix. Gel-based formulations also followed the Korsmeyer–Peppas model, suggesting non-Fickian diffusion as the main mechanism of drug release from the polymeric matrix.

### 2.8. Antibacterial Study

The present study evaluated the antimicrobial efficacy of CEO, AP, and their nanostructured formulations against key oral pathogens, including *Aggregatibacter actinomycetemcomitans* (*A. actinomycetemcomitan*), *Streptococcus mutans* (*S. mutans*), and *Eikenella corrodens* (*E. corrodens*). The results demonstrated that the CEO exhibited notable antibacterial activity across all the species tested. These findings are consistent with previous reports that attribute the antibacterial effect of CEO to its major component, EUG, which disrupts bacterial cell membranes and metabolic activity [50].

In contrast, free AP alone did not show measurable antimicrobial activity within the tested concentration range (>125 µg/mL). This is in line with the earlier literature reporting the limited bactericidal activity of AP, where its role has been suggested to be primarily anti-inflammatory or as a quorum-sensing inhibitor rather than being a direct antibacterial agent [51,52]. Notably, the NLC system combining the CEO and AP demonstrated enhanced antibacterial activity. While free AP did not exhibit measurable activity at the tested concentration, its co-delivery with CEO in the nanoparticles potentiated CEO’s effect. CEO disrupts bacterial membranes and targets DNA [53], while AP induces bacterial apoptosis via the activation of cellular oxidative pathways [54], together providing a multi-targeted mechanism that improves antibacterial efficacy, suggesting synergistic interactions. Our findings align with earlier studies reporting that AP can act synergistically with antibiotics to improve antibacterial outcomes [55].

The antibacterial efficacy of the developed formulations was evaluated using both MIC and MBC assays against *A. actinomycetemcomitans*, *S. mutans*, and *E. corrodens*. Table 5 summarizes the dilution levels and concentrations of CEO and AP (µg/mL) associated with bacterial growth inhibition and bactericidal effects (MIC and MBC). Among all formulations, NLC-AP (CEO + AP) demonstrated the highest antibacterial potency, exhibiting the lowest MIC values against *A. actinomycetemcomitan* (117.19 µg/mL of CEO; 1.4 µg/mL of AP) and *E. corrodens* (234 µg/mL of CEO; 3.9 µg/mL of AP) compared to NLC–CO, which showed higher MICs of 117.19 µg/mL and 468 µg/mL against both strains, respectively. The incorporation of NLC-AP into the P407 gel matrix resulted in increased MIC values as NP-5 showed MICs of CEO/AP: 234/3.9 µg/mL against *A. actinomycetemcomitans* and CEO/AP: 937.5/15.6 µg/mL against *E. corrodens*, while NP-10 further increased MICs to CEO/AP: 468/7.8 µg/mL against *A. actinomycetemcomitans*, indicating a formulation-dependent reduction in the apparent antibacterial activity.

The MBC analysis revealed that NLC-AP exerted a clear bactericidal effect against *A. actinomycetemcomitans* and *E. corrodens*, with MBC values of 468 µg/mL of CEO and 7.8 µg/mL of AP for both strains. In contrast, bactericidal concentrations increased progressively for gel-based systems, with NP-5 showing MBCs of CEO/AP: 1875/31.25 µg/mL and NP-10 requiring substantially higher concentrations (CEO/AP: 7500/125 µg/mL) to achieve complete bacterial eradication, possibly reflecting diffusion-limited antimicrobial activity within gel matrices. It is noteworthy, for *S. mutans*, that none of the tested formulations achieved bactericidal activity within the concentration range studied. The MBC values for *S. mutans* exceeded the highest tested concentrations (>7500 µg/mL CEO and >125 µg/mL AP), indicating a predominantly bacteriostatic effect, which is consistent with the higher MIC values observed for this strain across all formulations. The Supplementary File (Figure S3) presents images of the agar plates, which illustrate the MBC.

The reduced bactericidal efficacy observed after the incorporation of NLC-AP in the gelling system may be partially attributed to the increased viscosity of the formulation, which can restrict drug diffusion and microbial contact. However, at higher dilutions, the influence of viscosity is expected to be minimal, suggesting that viscosity alone cannot fully account for the observed increase in MIC values. Notably, in vitro drug release studies did not show much pronounced difference in AP release after P407 incorporation, indicating that the polymer did not significantly impede AP diffusion. However, CEO, which is embedded within the lipid matrix of NLC and serves as the primary contributor to the antimicrobial activity, may be affected by polymer–lipid interactions. DSC and Raman spectroscopic analyzes indicated weak but discernible interactions between the lipid components of NLC and P407. Such hydrophobic interactions can delay CEO diffusion from the nanocarriers or promote CEO–poloxamer micellar assembly formation, thus limiting CEO availability and ultimately hindering the antimicrobial effect. Overall, the reduced antibacterial activity of NLC-AP loaded gels cannot be attributed solely to viscosity-related diffusion limitations. Rather, weak physical polymer–lipid interactions may delay antimicrobial action or partially limit the availability of the key active component by restricting its diffusion or promoting transient sequestration within polymer–lipid assemblies. Consequently, the increased MIC values likely reflect a combination of delayed or modulated antimicrobial effects.

### 2.9. Morphology of Optimized Formulation

**Figure 7:**
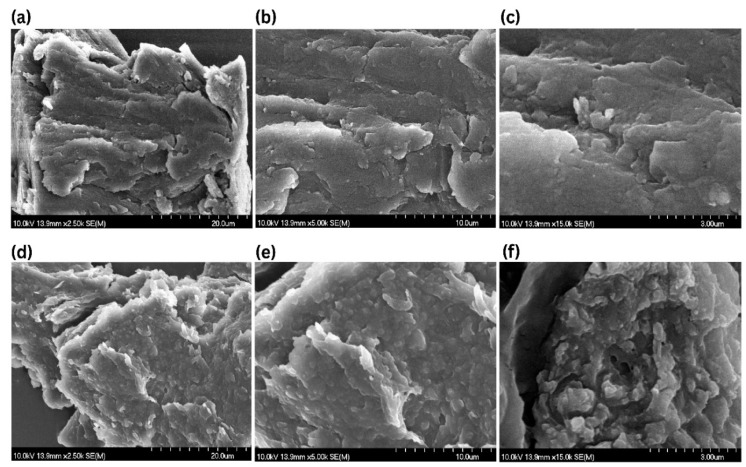
Morphological and structural analysis of the bank (**a**–**c**) and nanoloaded thermoresponsive gel (**d**–**f**) as examined by SEM. SEM micrographs were obtained at magnifications 2.5 K, 5 K, and 15 K.

The SEM micrographs of the lyophilized blank P407 gel are shown in Figure 7a–c, where it shows a dense, compact, and continuous polymeric network with a relatively smooth and layered morphology. Conversely, the nanoparticle-loaded P407 gel (NP-10), shown in Figure 7d–f, demonstrates the presence of nanoparticles uniformly embedded in the gel matrix. The nanoparticles are homogeneously distributed within the gelling system without noticeable aggregation, as observed across the different magnifications (20 µm, 10 µm, and 3 µm). This uniform dispersion indicates the effective incorporation of nanoparticles into the polymeric network while preserving the structural integrity of the gel.

### 2.10. Stability Study of Nanoparticles

**Figure 8:**
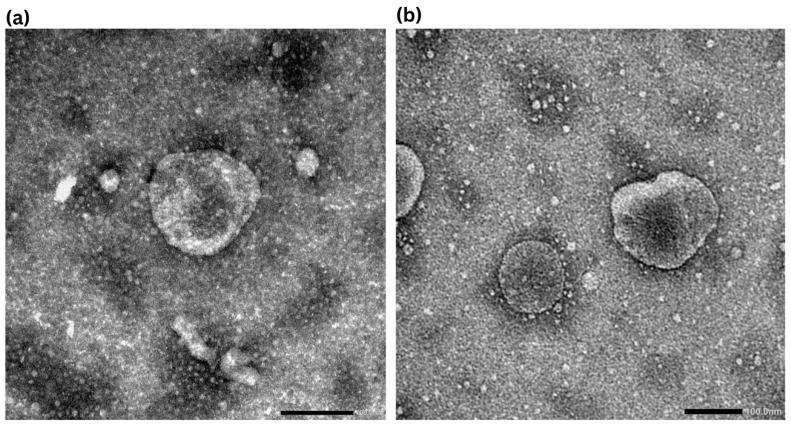
TEM images of the 10% P407 hydrogel loaded with NLC-AP (NP-10): (**a**) freshly prepared formulation showing uniformly distributed, nearly spherical nanoparticles, and (**b**) formulation after 9 months of storage, demonstrating preserved morphology and particle size without aggregation.

Based on the results of all the previous experiments, NP-10 was selected as the final optimized formulation due to its improved mucoadhesion, favorable rheology, and sustained drug release profile. Subsequently, this finalized formulation was subjected to further morphological and dispersity analysis over a period of 9 months using TEM. In Figure 8a (freshly prepared formulation), the nanoparticles appear predominantly semi-spherical to oval, with the particle size remaining in the average nanometer range (160 nm), indicating successful nanoparticle formation and their homogeneous dispersion within the gel matrix. In Figure 8b (after 9 months of storage), the nanoparticles almost retain their original morphology and size distribution, with no visible aggregation, fusion or particle collapse. A slight change in the shape of some nanoparticles is observed; however, this change is minimal and does not indicate structural instability. The absence of large clusters or irregular agglomerates confirms that the P407 hydrogel effectively stabilizes the NLC system during storage.

## 3. Conclusions

In the present study, NLC-based hydrogels were successfully developed as a platform for localized and sustained drug delivery. Integrating nanoparticles into P407 hydrogels allowed for controlled drug release without requiring high polymer concentrations. Among the tested formulations, NP-10 was identified as optimal, offering a balance of physicochemical stability, predictable drug release, and practical handling.

The formulation maintained the nanoscale dimensions, uniform particle distribution, and morphology even after 9 months, confirming structural and colloidal stability over the period of time. NP-10 exhibited sustained release governed by both diffusion and polymer relaxation, while rheological analysis demonstrated favorable viscoelastic and mucoadhesive properties. The system forms thermally stable gels at physiological temperature, and nanoparticle incorporation accelerates the gelation compared with blank hydrogels. Slight reductions in the in vitro antibacterial potency after gel incorporation likely reflect transient polymer–lipid interactions rather than a loss of efficacy.

Overall, these results demonstrate that NP-10 provides a stable, effective, and easily handled platform for the localized, controlled delivery of CEO and AP, highlighting the potential of NLC–hydrogel systems for therapeutic applications.

Given the biofilm-associated nature of periodontal pathogens, evaluating the formulation’s antibiofilm efficacy will be essential for the comprehensive assessment of its therapeutic potential. Additionally, mechanistic studies using relevant cell lines, followed by in vivo investigations, will be important to understand cellular responses and to validate the formulation’s translational and clinical relevance.

## 4. Materials and Methods

### 4.1. Materials

Compritol^®^ 888 ATO (glycerol dibehenate; SL (as solid lipid)), clove essential oil (CEO, derived from Eugenia species, containing 76.8% eugenol (EUG)), and Kolliphor^®^ RH40, KRH40, (PEG-40 hydrogenated castor oil; non-ionic surfactant) were sourced from Gattefossé (Saint-Priest Cedex, France), Sigma-Aldrich (Steinheim, Germany), and BASF SE Chemtrade GmbH (Ludwigshafen, Germany), respectively. Apigenin (AP) with a purity exceeding 94% and Poloxamer 407, P407 (Kolliphor^®^P407; containing 71.5–74.9% oxyethylene units), were obtained from Biosynth s.r.o. (Bratislava, Slovakia) and Sigma-Aldrich (St. Louis, MO, USA), respectively. Mucin (8% *v*/*v*, porcine gastric, and grade III) was provided by Sigma-Aldrich Co., Ltd. (Budapest, Hungary). Acetonitrile (AcN) and ethanol were acquired from VWR International (Debrecen, Hungary). All aqueous preparations were made using purified water passed through a Millipore Milli-Q^®^ 140 Gradient system (Merck Ltd., Budapest, Hungary). All the reagents and materials used were of analytical grade.

### 4.2. Preparation of a Nanoparticle-Loaded Polymeric Solution

Polymer-based sol-to-gel nanoformulations were developed by incorporating nanocarriers containing AP (NLC-AP) into P407 matrices. The NLC-AP nanoformulation was previously optimized using a Box–Behnken experimental design [15]. The optimized formulation consisted of 4.57% SL as the solid lipid, 1.5% CEO as the liquid lipid, and 3.57% KRH40 as the surfactant, with AP being incorporated at a concentration of 250 µg/mL. The nanoformulation was prepared by heating all the components to 85 °C, approximately 10 °C above the melting point of the solid lipid, followed by ultrasonic mixing to obtain a clear and homogeneous system. In the present study, polymeric sol-to-gel systems were prepared using varying concentrations of P407 (5%, 7%, 10%, and 15% *w*/*w*), as summarized in Table 6. Polymeric solutions were prepared using the cold method [20], wherein the required amount of P407 was gradually dispersed in the aqueous phase of the NLC, maintained at 4 °C under continuous magnetic stirring. The resulting dispersions loaded with or without NLC were then stored at 4 °C for 24 h to ensure complete polymer dissolution [56]. This procedure was employed for the preparation of both blank P407 solutions and P407 solutions incorporating NLC. Among all the samples, the 5% P407 solution (P-5) represented the lowest polymer concentration, while the 15% P407 solution (P-15) contained the highest polymer concentration and served as a reference for evaluating the effect of the increase in the polymer content on the physicochemical characterization of the formulation.

### 4.3. Physicochemical and Biopharmaceutical Characterization of Formulations

#### 4.3.1. Dynamic Light Scattering (DLS) of Nanoparticles

DLS was applied to determine the Z-average particle size (PS), polydispersity index (PDI), and zeta potential (ZP) of NLC-AP. These measurements were conducted using a Zetasizer Nano ZS instrument (Malvern Instruments, Worcestershire, UK). The diluted samples were weighed into capillary cuvettes, with the analysis performed at a controlled temperature of 25 °C. All the experiments were repeated three times (n = 3), and the data were reported as the mean values with ±SD.

#### 4.3.2. Morphological Analysis by Transmission Electron Microscopy (TEM)

The morphology and spatial arrangement of the prepared nanoformulation (NLC-AP) as well as nanoparticle-loaded gels were examined using TEM with a JEM-1400 Flash system (JEOL Ltd., Tokyo, Japan). The procedure was based on previously reported methods, with minor adjustments [57]. For negative staining, the dispersion was first diluted at a ratio of 1:100 using deionized water. A 10 µL aliquot of this diluted sample was then carefully poured onto a Formvar^®^-coated 150-mesh copper grid (Electron Microscopy Sciences, Hatfield, PA, USA). After allowing approximately one minute for adsorption, any excess liquid was gently removed with filter paper. To improve the image’s contrast, the grids were treated with a 2% uranyl acetate solution prepared in ultrapure water. This staining process was performed twice, with each step lasting about 2 min. The grids were subsequently left to dry overnight under a covered Petri dish to avoid contamination. The Initial observations were conducted at lower magnifications (5000×–20,000×) to evaluate overall morphology and particle distribution. Higher-magnification images (25,000×–50,000×) were then acquired using a 16-megapixel camera to capture finer structural details. Scale bars ranging from 100 to 200 nm were applied to the images for reference. Particle size analysis based on TEM images was performed using ImageJ software 1.54g (https://imagej.net/ij/index.html, accessed on 20 February 2025). To assess the morphological stability of nanoparticles within the gel matrices over time, another morphological analysis of optimized formulation was performed after 9 months.

#### 4.3.3. Determination of Entrapment Efficiency (EE%) and Drug Loading (DL%)

The EE% and DL% of the NLC-AP- and NLC-AP-based gels were determined by separating the unencapsulated AP from the drug delivery systems. This was achieved by centrifugation using Vivaspin 15R centrifugal filter units with a molecular weight cut-off of 5 kDa (Hydrosart™, Sartorius, Stonehouse, UK). Each formulation was subjected to centrifugation at 13,500 rpm for 30 min at 5 °C using a Hermle Z323K high-speed refrigerated centrifuge (HERMLE Labortechnik GmbH, Wehingen, Germany). The resulting filtrate, which contained free (non-entrapped) AP, was collected and analyzed using high-performance liquid chromatography (HPLC), as detailed in Section 4.3.8. The EE and DL were calculated using the following equations:

(1) $$EE\%=\frac{W_{loaded\;AP}\;-\;W_{free\;AP}}{W_{loaded\;AP}}\times 100$$

(2) $$DL\%=\frac{W_{loaded\;AP}-W_{free\;AP}}{W_{nanoparticles}}\times 100$$

#### 4.3.4. Differential Scanning Calorimetry (DSC)

Thermal analysis was carried out using a Mettler-Toledo DSC system (Mettler-Toledo GmbH, Gießen, Germany). The enthalpy measurements of SL and P407 were performed across a temperature range of 10 to 100 °C, applying a heating rate of 5K per min, with a continuous flow of nitrogen gas maintained at 40 mL/min [26]. The rest of all formulations were heated from 25 to 400 °C, with 10 °C/min heating rate and 50 mL/min nitrogen flow rate, method with slight modifications [58]. Approximately 2–5 mg of each sample was weighed and sealed in 40 µL aluminum crucibles for testing. All the recorded thermal events were normalized to the sample mass. The data processing and analysis was performed using STARe Software, version 9.00 (Mettler-Toledo GmbH, Gießen, Germany).

#### 4.3.5. Raman Spectrum and Chemical Mapping

Raman spectroscopy was conducted using a DXR Dispersive Raman Spectrometer (Thermo Fisher Scientific Inc., Waltham, MA, USA) to investigate possible interactions among the additives, AP, NLC-AP, and polymeric systems loaded with NLC-AP. The system was equipped with a charge-coupled device (CCD) detector and employed a diode laser operating at 780 nm, covering a spectral range from 3000 to 200 cm^−1^. Spectral acquisition was carried out using a 24 mW laser through a 25 µm slit aperture, targeting a focal spot of approximately 2–3 µm in size. The instrument provided a spectral resolution between 2.4 and 4.4 cm^−1^. Each sample spectrum was captured with a 6 s exposure time, repeated 24 times to enhance the signal’s clarity. Spectral data were collected and analyzed using OMNIC™ 8.2 software tailored for Dispersive Raman software 8.3.104 package (Thermo Fisher Scientific Inc., Waltham, MA, USA). To ensure consistency and comparability, the acquired spectra were normalized to compensate for any intensity fluctuations at different measurement points.

Confocal Raman microscopy is a powerful label-free technique that enables chemical-specific spatial mapping, making it ideal for characterizing multicomponent systems by assessing drug distribution and homogeneity within the formulation [59]. Raman chemical mapping was employed to visualize the localization of AP and CEO within the gelling formulation. Samples were mounted on glass slides covered with aluminum foil, which served as sample holders. Raman imaging was performed using a DXR Raman Microscope (Thermo Fisher Scientific Inc., Waltham, MA, USA), equipped with a charge-coupled device (CCD) camera for data capture. The Raman map was recorded at 50× magnification over an area of 100 × 1000 µm with a spatial resolution of 50 µm between 66 spectral points with 10 µm step size. A 780 nm diode laser operating at 24 mW power, along with a 50 µm slit aperture, was utilized during the mapping process. Each spectrum was acquired with an individual scan duration of more than 2 s, following an initial 2 s laser exposure, and represented an average of 32 accumulations across the spectral range of 3300 to 200 cm^−1^. The signal’s preprocessing included corrections for both cosmic rays and background fluorescence to enhance the signal quality. To identify the presence and spatial distribution of AP, CEO, and additives, their characteristic Raman spectrum served as the reference. The data interpretation and Raman image generation were performed using OMNIC™ 8.2 software (Thermo Fisher Scientific Inc., Waltham, MA, USA).

#### 4.3.6. Rheological Study

Rheological evaluation of all the samples was performed with Physica MCR 302 Modular Compact Rheometer (Anton Paar, Graz, Austria). A cone and plate-type measuring device, CP25 of 25 mm diameter (cone angle: 1°; gap at the middle of the cone: 0.05 mm) was used in the oscillatory mode. Prior to temperature sweep testing, each sample was loaded between the plates and allowed to equilibrate at the starting temperature (15 °C). The gelation temperature measurements were conducted at a constant frequency (1 rad//min) and strain (1%), and the temperature was gradually increased to 40 °C at a controlled ramp rate of 1 °C/min. The point at which G′ and G″ intersect, representing the sol–gel transition, is widely accepted as a key indicator of the phase change in thermoresponsive hydrogels. The gelation time (time-ramp) was recorded at 37 °C, as the crossover point of both the G′ and G″ curves with constant frequency (1 rad/min) and strain (1%). Following the time ramp assessment, the frequency sweep measurements were continuously recorded in the angular frequency range of 0.1 to 100 rad/s at 37 °C.

#### 4.3.7. Evaluation of Mucoadhesive Properties

Mucoadhesive performance was assessed using a Texture Analyzer (Stable Micro Systems Ltd., Godalming, Surrey, UK) equipped with a 5 kg load cell. A filter paper moistened with 50 µL of 8% *w*/*w* mucin solution was placed on the mucoadhesion test probe. Throughout the test, both the assembly and the model mucosal surface were maintained at 37 °C. A 20 µL sample was positioned on a cylindrical probe (10 mm in diameter). The probe was brought into contact with the mucosal surface under a constant preload force of 2500 mN and held in place for 3 min to establish adhesion. Detachment was then initiated by moving the probe upward at a speed of 2.5 mm/min to disrupt the adhesive interaction. Each sample was tested in quintuplicate to determine the adhesive force and the work of adhesion, following the protocols described by Ashfaq et al. [60].

#### 4.3.8. Release Study and Kinetics

The in vitro release profiles of AP were evaluated from both NLC and the corresponding gel formulations under identical conditions using a dialysis membrane technique to assess the drug release behavior and formulation performance. For each formulation, triplicate samples (0.7 g) were loaded into Spectra/Por® 4 dialysis tube (Spectrum Laboratories, Inc., Rancho Dominguez, CA, USA) with a molecular weight cut-off (MWCO) of 12–14 kDa, which was 6.37 mm in diameter and 10 mm in width. The release medium consisted of a phosphate-buffered saline (PBS)–ethanol mixture (1:1 *v*/*v*, pH 7.4) with a total volume of 10.5 mL maintained at 37 °C. The study was carried out in closed vials for 48 h and sampling was performed at ten predetermined intervals (0.5, 1, 2, 6, 12, 18, 24, 36, 42, and 48 h). At each time point, 0.3 mL of the release medium was withdrawn and replaced with an equal volume of fresh PBS–ethanol solution to maintain consistent volume and sink conditions. To prevent evaporation, the setup was sealed throughout the experiment.

For the quantification of AP, chromatographic analysis was performed using a SHIMADZU Nexera X2 ultra-high-performance liquid chromatography (UHPLC) system (Shimadzu Corporation, Kyoto, Japan). Separation was achieved on a Phenomenex Kinetex C18 reverse phase column (150 × 4.6 mm; 2.5 µm particle size) maintained at 40 °C. The mobile phase consisted of two components: (A) acidified water (pH 4.5, adjusted with phosphoric acid) and (B) AcN. A gradient elution was applied, starting at 20% of solvent B, reaching up to 70% over 9 min and later returned to the initial 20%. A constant flow rate of 0.5 mL/min was maintained over a 12 min analytical run. AP was detected at a wavelength of 337 nm using a diode array UV-VIS detector, with a retention time of 8.3 min. The LOD and LOQ were 1.4 µg/mL and 4.5 µg/mL, respectively. The cumulative drug release (µg) was calculated and plotted as a function of time (h). Additionally, the release kinetics of AP from the formulations were analyzed using the DDSolver (MS Excel add-in software package) mathematical models to understand the underlying drug release mechanisms.

#### 4.3.9. Morphological Analysis of Nanoparticle-Loaded Gel by SEM

The surface morphology of the optimized formulation was examined using SEM with a Hitachi S4700 system (Hitachi Scientific Ltd., Tokyo, Japan), operating at an accelerating voltage of 10 kV. Prior to the imaging, the samples were coated with a thin layer of gold–palladium using a Bio-Rad SC 502 sputter coater (VG Microtech, Uckfield, UK) for 150 s. The chamber pressure was maintained between 1.3 and 13.0 mPa. SEM imaging was performed using an accelerating voltage of 10 kV and a current of 10 mA, maintaining a working distance of 13.9 mm. The micrographs were captured at magnifications of 2500×, 5000×, and 15,000×.

#### 4.3.10. Antibacterial Study with Different Bacterial Strains

The antibacterial efficacy of the investigated formulations was assessed using *Aggregatibacter actinomycetemcomitans* DSM 11122 (*A*. *actinomycetemcomitan*), *Streptococcus mutans* ATCC 25175 (*S. mutans*), and *Eikenella corrodens* ATCC 23834 (*E. corrodens*) as representative reference strains. All three microorganisms were propagated on Columbia agar supplemented with 5% (*v*/*v*) sheep blood (bioMérieux, Marcy-l’Étoile, France) and incubated at 35 °C under a 5% CO_2_-enriched atmosphere for 24 h. Bacterial inocula were prepared by suspending freshly grown colonies in sterile 0.85% (*w*/*v*) sodium chloride solution, and the turbidity was standardized to 0.5 McFarland, corresponding to approximately 1 × 10^8^ CFU/mL.

##### Minimum Inhibitory Concentration (MIC)

The MIC values of the free and encapsulated formulations were determined using a broth microdilution method in 96-well microplates, according to the European Committee on Antimicrobial Susceptibility Testing (EUCAST) guidelines. Serial dilutions of each formulation of NLC-AP, NLC-AP-loaded gels, and NLC–CO (NLC without AP) were prepared using distilled water as the diluent. The AP suspension and the CO emulsion were prepared in buffer with 1% KRH40 to ensure the homogeneity of the system. Subsequently, 100 µL of bacterial suspension (≈1 × 10^5^ CFU/mL) prepared in brain heart infusion (BHI) broth (Oxoid, Basingstoke, UK) was added to each well.

In the case of CEO, two-fold serial dilutions yielded final concentrations ranging from 15,000 to 29.3 µg/mL. For AP the concentration ranges from 250 to 0.35 µg/mL. Distilled water served as the negative control. To account for the inherent turbidity of the nanoparticulate systems, each dilution was mixed with BHI broth alone as a background control. The positive controls consisted of BHI broth inoculated with bacterial suspension and chlorhexidine, tested in a concentration range of 0.008–0.25 µg/mL using two-fold serial dilutions. All the microplates were incubated at 37 °C under 5% CO_2_ for 24 h, after which the bacterial growth was quantified by measuring the optical density at 620 nm. All the assays were performed in triplicate.

##### Minimum Bactericidal Concentration (MBC)

The MBC, defined as the lowest concentration capable of achieving complete bacterial killing, was determined by subculturing 10 µL aliquots from wells corresponding to the MIC assay onto Columbia agar plates supplemented with 5% sheep blood (bioMérieux, Marcy-l’Étoile, France). The plates were incubated at 35 °C in a 5% CO_2_ atmosphere for 24 h, and the absence of visible bacterial growth was considered indicative of bactericidal activity.

#### 4.3.11. Stability Study of Nanoparticles Within the Gelling System

To assess the physicochemical stability of the nanoparticles, the optimized thermoresponsive gel was stored in airtight glass vials in refrigerated conditions (4 ± 2 °C). The samples were stored under the same conditions for 9 months to evaluate the particle morphology, the uniformity of distribution, and the presence of any aggregation. TEM was performed as described in Section 4.3.2. Collectively, these assessments provided comprehensive insight into the structural and colloidal stability of the nanoparticles within the system over time.

#### 4.3.12. Statistical Analysis

All the experiments were conducted in triplicate and the results are expressed as the mean values ± SD, where applicable. The data visualization and the statistical evaluations were performed using GraphPad Prism version 8.0.2 (GraphPad Software, Inc., La Jolla, CA, USA) and OriginPro^®^ version 8.6 (OriginLab Corporation, Northampton, MA, USA). The statistical significance between two groups was determined using Student’s *t* test, while comparisons between multiple groups were performed using one-way analysis of variance (ANOVA) followed by Tukey’s post hoc test to account for multiple comparisons.

## Figures and Tables

**Table 1:** Physical parameters of apigenin-loaded NLC.

Formulation	Solid Lipid (%)	Liquid Lipid (%)	Surfactant (%)	Apigenin (µg/mL)	PS (nm)	PDI	ZP (mV)	EE (%)	DL (%)
NLC-AP	4.57	1.50	3.57	250	188.5 ± 2.2	0.25 ± 0.03	−17 ± 2.0	95.3 ± 2.5	4.7 ± 0.3

**Table 2:** EE and DL of NLC-AP at different concentrations of poloxamer gels.

Formulation	EE (%)	DL (%)
NP-5	97.3 ± 2.3	4.8 ± 0.7
NP-7	96.9 ± 1.5	4.7 ± 0.5
NP-10	98.0 ± 2.8	4.9 ± 0.9
NP-15	97.8 ± 2.7	4.8 ± 0.8

**Table 3:** Gelling properties of blank and loaded in situ gelling systems. Values are presented as the mean ± standard deviation (SD), n = 3.

Formulations	Gelling Temperature (℃)	Gelling Time at 37 °C (min)	G′ at 10 rad/s (Pa)	G′ at 1 rad/s (Pa)	tanδ at 10 rad/s (-)	tanδ at 1 rad/s (-)
NP-5	29.84 ± 0.98	1.4 ± 0.86	3703.6 ± 0.05	3736.7 ± 0.09	0.081 ± 0.05	0.169 ± 0.07
P-5	32.84 ± 1.25	5.8 ± 0.02	1191.3 ± 0.30	1341.3 ± 1.01	0.052 ± 0.01	0.180 ± 0.04
NP-7	30.64 ± 0.71	1.0 ± 0.82	4620.4 ± 0.40	4072.5 ± 0.09	0.075 ± 0.07	0.159 ± 0.15
P-7	32.04 ± 1.50	3.3 ± 0.04	3486.1 ± 0.50	3717.9 ± 0.99	0.030 ± 0.02	0.030 ± 0.05
NP-10	27.24 ± 2.01	<1	4962.3 ± 0.35	5233.6 ± 0.07	0.058 ± 0.04	0.129 ± 0.09
P-10	28.64 ± 2.05	2.4 ± 0.10	2424.8 ± 0.33	2501.3 ± 0.88	0.064 ± 0.06	0.183 ± 0.12
NP-15	<15	gel state	14468 ± 0.03	12874 ± 0.10	0.060 ± 0.03	0.187 ± 0.10
P-15	22.87 ± 2.10	1.0 ± 0.5	7969.8 ± 0.10	6465.1 ± 2.09	0.131 ± 0.07	0.240 ± 0.61

**Table 4:** Release kinetic models presenting the release pattern of nanoparticle-loaded and unloaded gels in comparison to suspension.

Release Models	Equations	NLC-AP R^2^	NP-5 R^2^	NP-7 R^2^	NP-10 R^2^	NP-15 R^2^	AP-Suspension R^2^
Zero order	F = k0 × t	0.693	0.795	0.781	0.806	0.864	0.866
First order	F = 100 × [1 − Exp(−k1 × t)]	0.825	0.888	0.876	0.893	0.926	0.942
Higuchi	F = kH × t^0.5	0.951	0.982	0.965	0.977	0.986	0.978
Korsmeyer–Peppas	F = kKP × t^n	0.979	0.989	0.989	0.985	0.998	0.971
*n*	-	0.43	0.49	0.48	0.55	0.50	0.57

**Table 5:** Dilution levels and concentrations of bioactive corresponding to MIC and MBC for the tested formulations against selected oral pathogens.

Samples	*A. actinomycetemcomitans*		*S. mutans*		*E. corrodens*	
	MIC	MBC	MIC	MBC	MIC	MBC
	Dilutions/Concentration of CEO (µg/mL)/Concentration of AP (µg/mL)					
Free CEO	1:64/234	1:16/937	1:16/937	1:2/7500	1:32/468	1:16/937
Free AP	>1:2/>125	>1:2/>125	>1:2/>125	>1:2/>125	>1:2/>125	>1:2/>125
NLC–CO	1:128/117/-	1:16/937/-	1:16/937/-	>1:2/>7500/-	1:32/468/-	1:16/937/-
NLC-AP	1:128/117/1.4	1:32/468/7.8	1:32/468/7.8	>1:2/>7500/>125	1:64/234/3.9	1:32/468/7.8
NP-5	1:64/234/3.9	1:8/1875/31.2	1:8/1875/31.2	>1:2/>7500/>125	1:16/937/15.6	1:8/1875/31.2
NP-10	1:32/468/7.8	1:2/7500/125	1:4/3750/62.5	>1:2/>7500/>125	1:16/937/15.6	1:2/7500/125
P407	>1:2	>1:2	>1:2	>1:2	>1:2	>1:2

**Table 6:** Composition of nanoparticle-loaded solutions alongside corresponding blank polymeric formulations to compare the influence of nanoparticle incorporation.

Formulations	P407 (% *w*/*w*)	NLC-AP (%)	Water (%)
NP-5	5	70	25
NP-7	7	70	23
NP-10	10	70	20
NP-15	15	70	15
P-5 *	5	-	95
P-7 *	7	-	93
P-10 *	10	-	90
P-15 *	15	-	85

* Samples for structural comparison.

## Data Availability

Data are contained within the article.

## Supplementary Materials

The following supporting information can be downloaded at https://www.mdpi.com/article/10.3390/gels12040268/s1. Figure S1: Microscopic images of surfaces subjected to Raman mapping: (a) NLC–CO, (b) NLC-AP, (c) NP-10. Figure S2: Temperature and time sweep test graphs of nanoloaded and blank poloxamer gels. Figure S3: Antibacterial study results against three bacterial strains on agar media.

## References

[1] Ashfaq R., Kovács A., Berkó S., Budai-Szűcs M. (2024). Developments in Alloplastic Bone Grafts and Barrier Membrane Biomaterials for Periodontal Guided Tissue and Bone Regeneration Therapy. Int. J. Mol. Sci..

[2] Cekici A., Kantarci A., Hasturk H., Van Dyke T.E. (2014). Inflammatory and immune pathways in the pathogenesis of periodontal disease. Periodontology 2000.

[3] Łasica A., Golec P., Laskus A., Zalewska M., Gędaj M., Popowska M. (2024). Periodontitis: Etiology; conventional treatments, and emerging bacteriophage and predatory bacteria therapies. Front. Microbiol..

[4] Asghar S., Khan I.U., Salman S., Khalid S.H., Ashfaq R., Vandamme T.F. (2021). Plant-derived nanotherapeutic systems to counter the overgrowing threat of resistant microbes and biofilms. Adv. Drug Deliv. Rev..

[5] Ashfaq R., Rasul A., Asghar S., Kovács A., Berkó S., Budai-Szűcs M. (2023). Lipid Nanoparticles: An Effective Tool to Improve the Bioavailability of Nutraceuticals. Int. J. Mol. Sci..

[6] Nisar M.F., Khadim M., Rafiq M., Chen J., Yang Y., Wan C.C. (2021). Pharmacological Properties and Health Benefits of Eugenol: A Comprehensive Review. Oxid. Med. Cell. Longev..

[7] Abdou A., Ennaji H., Maaghloud F.E., Azhary K.E., Badou A., Elmakssoudi A., Aboulmouhajir A., Ibenmoussa S., JamalEddine J., Dakir M. (2024). In silico and in vivo anti-inflammatory effect of eugenol and acetyleugenol. Sci. Afr..

[8] Guenette S.A., Beaudry F., Marier J.F., Vachon P. (2006). Pharmacokinetics and anesthetic activity of eugenol in male Sprague–Dawley rats. J. Vet. Pharmacol. Ther..

[9] Jindal A., Kumar A. (2022). Physical characterization of clove oil based self Nano-emulsifying formulations of cefpodoxime proxetil: Assessment of dissolution rate, antioxidant & antibacterial activity. OpenNano.

[10] Singh D., Kumari K., Ahmed S. (2022). Natural herbal products for cancer therapy. Understanding Cancer.

[11] Huang H., Han J., Liu Y., Zhang Q., Zhou Y., Zheng S., Zhou C., Bao C., Qing C., Lu W. (2025). Exploring the molecular mechanism of apigenin in treating bronchiectasis based on network pharmacology and molecular docking. Sci. Rep..

[12] Allemailem K.S., Almatroudi A., Alharbi H.O.A., AlSuhaymi N., Alsugoor M.H., Aldakheel F.M., Khan A.A., Rahmani A.H. (2024). Apigenin: A Bioflavonoid with a Promising Role in Disease Prevention and Treatment. Biomedicines.

[13] Jeong G.-S., Lee S.-H., Jeong S.-N., Kim Y.-C., Kim E.-C. (2009). Anti-inflammatory effects of apigenin on nicotine- and lipopolysaccharide-stimulated human periodontal ligament cells via heme oxygenase-1. Int. Immunopharmacol..

[14] Kashyap P., Shikha D., Thakur M., Aneja A. (2022). Functionality of apigenin as a potent antioxidant with emphasis on bioavailability, metabolism, action mechanism and in vitro and in vivo studies: A review. J. Food Biochem..

[15] Ashfaq R., Kovács A., Berkó S., Katona G., Paróczai D., Szécsényi M., Burián K., Vályi P., Veres K., Girst G. (2026). Co-encapsulated apigenin and clove oil in nanostructured lipid carriers for enhanced periodontal disease therapy. J. Drug Deliv. Sci. Technol..

[16] Agrawal A.K., Das M., Jain S. (2012). In situ gel systems as ‘smart’ carriers for sustained ocular drug delivery. Expert Opin. Drug Deliv..

[17] Ruel-Gariépy E., Leroux J.-C. (2004). In situ-forming hydrogels—Review of temperature-sensitive systems. Eur. J. Pharm. Biopharm..

[18] Ashfaq R., Kovács A., Berkó S., Budai-Szűcs M. (2025). Smart biomaterial gels for periodontal therapy: A novel approach. Biomed. Pharmacother..

[19] Agnello S., Gasperini L., Mano J.F., Pitarresi G., Palumbo F.S., Reis R.L., Giammona G. (2017). Synthesis, mechanical and thermal rheological properties of new gellan gum derivatives. Int. J. Biol. Macromol..

[20] Brambilla E., Locarno S., Gallo S., Orsini F., Pini C., Farronato M., Thomaz D.V., Lenardi C., Piazzoni M., Tartaglia G. (2022). Poloxamer-Based Hydrogel as Drug Delivery System: How Polymeric Excipients Influence the Chemical-Physical Properties. Polymers.

[21] Dewan M., Adhikari A., Jana R., Chattopadhyay D. (2023). Development, evaluation and recent progress of ocular in situ gelling drug delivery vehicle based on poloxamer 407. J. Drug Deliv. Sci. Technol..

[22] Zięba M., Chaber P., Duale K., Martinka Maksymiak M., Basczok M., Kowalczuk M., Adamus G. (2020). Polymeric Carriers for Delivery Systems in the Treatment of Chronic Periodontal Disease. Polymers.

[23] Morelli L., Cappelluti M.A., Ricotti L., Lenardi C., Gerges I. (2017). An Injectable System for Local and Sustained Release of Antimicrobial Agents in the Periodontal Pocket. Macromol. Biosci..

[24] Mansuri S., Kesharwani P., Jain K., Tekade R.K., Jain N.K. (2016). Mucoadhesion: A promising approach in drug delivery system. React. Funct. Polym..

[25] Giuliano E., Paolino D., Fresta M., Cosco D. (2018). Mucosal Applications of Poloxamer 407-Based Hydrogels: An Overview. Pharmaceutics.

[26] Ashfaq R., Tóth N., Kovács A., Berkó S., Katona G., Ambrus R., Polgár T.F., Burián K., Szécsényi M., Budai-Szűcs M. (2025). Hydrogel–Nanolipid Formulations for the Complex Anti-Inflammatory and Antimicrobial Therapy of Periodontitis. Pharmaceutics.

[27] Ortiz A.C., Yañez O., Salas-Huenuleo E., Morales J.O. (2021). Development of a Nanostructured Lipid Carrier (NLC) by a Low-Energy Method, Comparison of Release Kinetics and Molecular Dynamics Simulation. Pharmaceutics.

[28] Rosiak N., Tykarska E., Miklaszewski A., Pietrzak R., Cielecka-Piontek J. (2025). Enhancing the Solubility and Dissolution of Apigenin: Solid Dispersions Approach. Int. J. Mol. Sci..

[29] Huang Y., Zu Y., Zhao X., Wu M., Feng Z., Deng Y., Zu C., Wang L. (2016). Preparation of inclusion complex of apigenin-hydroxypropyl-β-cyclodextrin by using supercritical antisolvent process for dissolution and bioavailability enhancement. Int. J. Pharm..

[30] Shakeel F., Alshehri S., Ibrahim M.A., Elzayat E.M., Altamimi M.A., Mohsin K., Alanazi F.K., Alsarra I.A. (2017). Solubility and thermodynamic parameters of apigenin in different neat solvents at different temperatures. J. Mol. Liq..

[31] Xu X., Zhao C., Yang H., Jian Y., Zhang Z.-R., Huang Y. (2011). Anti-inflammatory activity of injectable dexamethasone acetate-loaded nanostructured lipid carriers. Drug Deliv..

[32] Ibrahim M., EL-Badry M., Hassan M., Elsaghir H. (2013). Performance of Poloxamer 407 as Hydrophilic Carrier on the Binary Mixtures with Nimesulide. Farmacia.

[33] Hogarth C., Arnold K., McLauchlin A., Rannard S.P., Siccardi M., McDonald T.O. (2021). Evaluating the impact of systematic hydrophobic modification of model drugs on the control, stability and loading of lipid-based nanoparticles. J. Mater. Chem. B.

[34] Bodratti A.M., Alexandridis P. (2018). Formulation of Poloxamers for Drug Delivery. J. Funct. Biomater..

[35] Corredor C., Teslova T., Cañamares M.V., Chen Z., Zhang J., Lombardi J.R., Leona M. (2009). Raman and surface-enhanced Raman spectra of chrysin, apigenin and luteolin. Vib. Spectrosc..

[36] Vargas Jentzsch P., Gualpa F., Ramos L.A., Ciobotă V. (2018). Adulteration of clove essential oil: Detection using a handheld Raman spectrometer. Flavour Fragr. J..

[37] Perez N., Altube M.J., Barbosa L.R.S., Romero E.L., Perez A.P. (2022). *Thymus vulgaris* essential oil + tobramycin within nanostructured archaeolipid carriers: A new approach against *Pseudomonas aeruginosa* biofilms. Phytomedicine.

[38] Gouadec G., Bellot-Gurlet L., Baron D., Colomban P. (2012). Raman Mapping for the Investigation of Nano-phased Materials. Raman Imaging: Techniques and Applications.

[39] Escobar-Chávez J.J., López-Cervantes M., Naik A., Kalia Y., Quintanar-Guerrero D., Ganem-Quintanar A. (2006). Applications of thermo-reversible pluronic F-127 gels in pharmaceutical formulations. J. Pharm. Pharm. Sci..

[40] Po J.M.C., Kieser J.A., Gallo L.M., Tésenyi A.J., Herbison P., Farella M. (2011). Time-frequency analysis of chewing activity in the natural environment. J. Dent. Res..

[41] Tanislav A.E., Pușcaș A., Păucean A., Mureșan A.E., Semeniuc C.A., Mureșan V., Mudura E. (2022). Evaluation of Structural Behavior in the Process Dynamics of Oleogel-Based Tender Dough Products. Gels.

[42] Zhang G., Zhang Z., Sun M., Yu Y., Wang J., Cai S. (2022). The Influence of the Temperature on the Dynamic Behaviors of Magnetorheological Gel. Adv. Eng. Mater..

[43] Diaz-Salmeron R., Toussaint B., Huang N., Bourgeois Ducournau E., Alviset G., Goulay Dufaÿ S., Hillaireau H., Dufaÿ Wojcicki A., Boudy V. (2021). Mucoadhesive Poloxamer-Based Hydrogels for the Release of HP-β-CD-Complexed Dexamethasone in the Treatment of Buccal Diseases. Pharmaceutics.

[44] Khan T.U., Mushtaq N., Iqbal T., Liu C.-G. (2026). Mucoadhesive hyaluronic acid composites for prolonged gastric retention. Int. J. Pharm..

[45] Szafraniec J., Antosik A., Knapik-Kowalczuk J., Chmiel K., Kurek M., Gawlak K., Odrobińska J., Paluch M., Jachowicz R. (2019). The Self-Assembly Phenomenon of Poloxamers and Its Effect on the Dissolution of a Poorly Soluble Drug from Solid Dispersions Obtained by Solvent Methods. Pharmaceutics.

[46] Swain G.P., Patel S., Gandhi J., Shah P. (2019). Development of Moxifloxacin Hydrochloride loaded in-situ gel for the treatment of periodontitis: In-vitro drug release study and antibacterial activity. J. Oral Biol. Craniofacial Res..

[47] Dabhi M.R., Nagori S.A., Gohel M.C., Parikh R.K., Sheth N.R. (2010). Formulation development of smart gel periodontal drug delivery system for local delivery of chemotherapeutic agents with application of experimental design. Drug Deliv..

[48] Amarachi C.S., Onunkwo G., Onyishi I. (2013). Kinetics and mechanisms of drug release from swellable and non swellable matrices: A review. Res. J. Pharm. Biol. Chem. Sci..

[49] Zhu W., Long J., Shi M. (2023). Release Kinetics Model Fitting of Drugs with Different Structures from Viscose Fabric. Materials.

[50] Jeyakumar G.E., Lawrence R. (2021). Mechanisms of bactericidal action of Eugenol against *Escherichia coli*. J. Herb. Med..

[51] Pei Z.-J., Li C., Dai W., Lou Z., Sun X., Wang H., Khan A.A., Wan C. (2023). The Anti-Biofilm Activity and Mechanism of Apigenin-7-O-Glucoside Against *Staphylococcus aureus* and *Escherichia coli*. Infect. Drug Resist..

[52] Alum E.U., Gulumbe B.H., Izah S.C., Uti D.E., Aja P.M., Igwenyi I.O., Offor C.E. (2025). Natural product-based inhibitors of quorum sensing: A novel approach to combat antibiotic resistance. Biochem. Biophys. Rep..

[53] Li J., Li C., Shi C., Aliakbarlu J., Cui H., Lin L. (2022). Antibacterial mechanisms of clove essential oil against *Staphylococcus aureus* and its application in pork. Int. J. Food Microbiol..

[54] Kim S., Woo E.-R., Lee D.G. (2020). Apigenin promotes antibacterial activity via regulation of nitric oxide and superoxide anion production. J. Basic Microbiol..

[55] Akilandeswari K., Ruckmani K. (2016). Synergistic antibacterial effect of apigenin with Î^2^-lactam antibiotics and modulation of bacterial resistance by a possible membrane effect against methicillin resistant *Staphylococcus aureus*. Cell. Mol. Biol..

[56] Menshutina N., Derkach V., Mokhova E., Gordienko M., Menshutina N., Derkach V., Mokhova E., Gordienko M. (2025). Investigation of Rheological Characteristics of Thermosensitive Nasal In Situ Gels Based on P407 and Their Effect on Spray Pattern. Gels.

[57] Correia A.C., Costa I., Silva R., Sampaio P., Moreira J.N., Sousa Lobo J.M., Silva A.C. (2024). Design of experiment (DoE) of mucoadhesive valproic acid-loaded nanostructured lipid carriers (NLC) for potential nose-to-brain application. Int. J. Pharm..

[58] Aldawsari M., Ahmed M., Fatima F., Anwer M.K., Katakam P., Khan A. (2021). Development and Characterization of Calcium-Alginate Beads of Apigenin: In Vitro Antitumor, Antibacterial, and Antioxidant Activities. Mar. Drugs.

[59] Breitenbach J., Schrof W., Neumann J. (1999). Confocal Raman-Spectroscopy: Analytical Approach to Solid Dispersions and Mapping of Drugs. Pharm. Res..

[60] Ashfaq R., Sisa B., Kovács A., Berkó S., Szécsényi M., Burián K., Vályi P., Budai-Szűcs M. (2023). Factorial design of in situ gelling two-compartment systems containing chlorhexidine for the treatment of periodontitis. Eur. J. Pharm. Sci. Off. J. Eur. Fed. Pharm. Sci..

