# Supplementary material for: Engineering Thermoresponsive In Situ Gels Incorporating Nutraceutical-Laden Nanostructured Lipid Carriers for Controlled Periodontal Drug Release
Source: Gels. 2026 Mar 24;12(4):268. doi: 10.3390/gels12040268 (PMC13115488; doi:10.3390/gels12040268)

Engineering Thermoresponsive In Situ Gels Incorporating Nutraceutical-Laden Nanostructured Lipid Carriers for Controlled Periodontal Drug Release

Rabia Ashfaq [1], Anita Kovács [1], Szilvia Berkó [1], Gábor Katona [1], Rita Ambrus [1], Tamás Ferenc Polgár [2,3], Mária Szécsényi [4], Katalin Burián [4] and Mária Budai-Szűcs [1,*]

[1] Institute of Pharmaceutical Technology and Regulatory Affairs, Faculty of Pharmacy, University of Szeged, H-6720 Szeged, Hungary; rabia.ashfaq@szte.hu (R.A.); gasparne.kovacs.anita@szte.hu (A.K.); berko.szilvia@szte.hu (S.B.); katona.gabor@szte.hu (G.K.); ambrus.rita@szte.hu (R.A.)

[2] Core Facility, HUN-REN Biological Research Centre, H-6726 Szeged, Hungary; polgar.tamas@brc.hu

[3] HUN-REN-SZTE Neuroscience Research Group, Hungarian Research Network, University of Szeged, H-6725 Szeged, Hungary

[4] Department of Medical Microbiology, University of Szeged, H-6720 Szeged, Hungary; szecsenyi.maria@med.u-szeged.hu (M.S.); burian.katalin@med.u-szeged.hu (K.B.)

* Correspondence: budai-szucs.maria@szte.hu; Tel.: +36-62545573

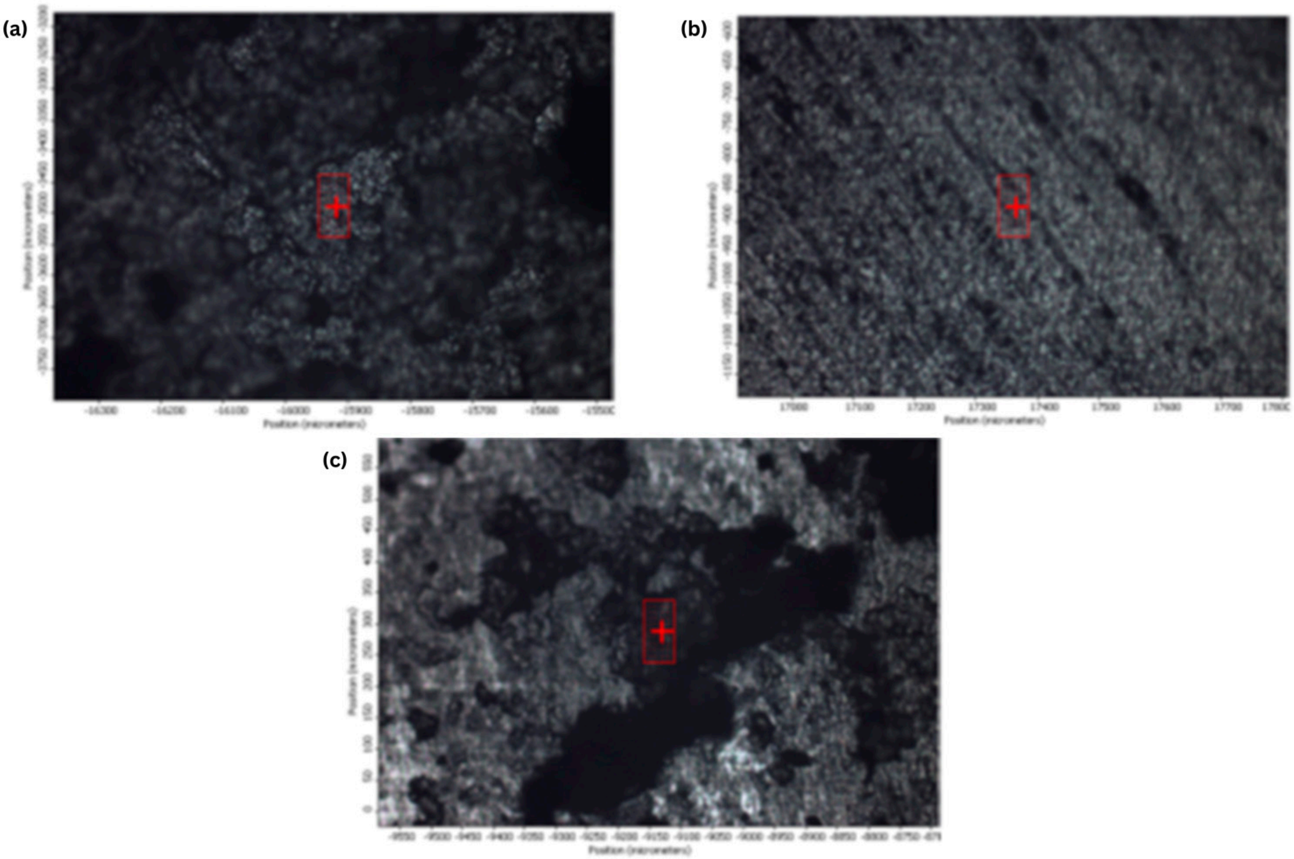

Figure S1. Microscopic images of surfaces subjected to Raman mapping (a) NLC–CO, (b) NLC-AP, (c) NP-10.

Figure S2 (a-h): Temperature and time sweep test graphs of nanoloaded and blank poloxamer gels

Temperature–Sweep Study

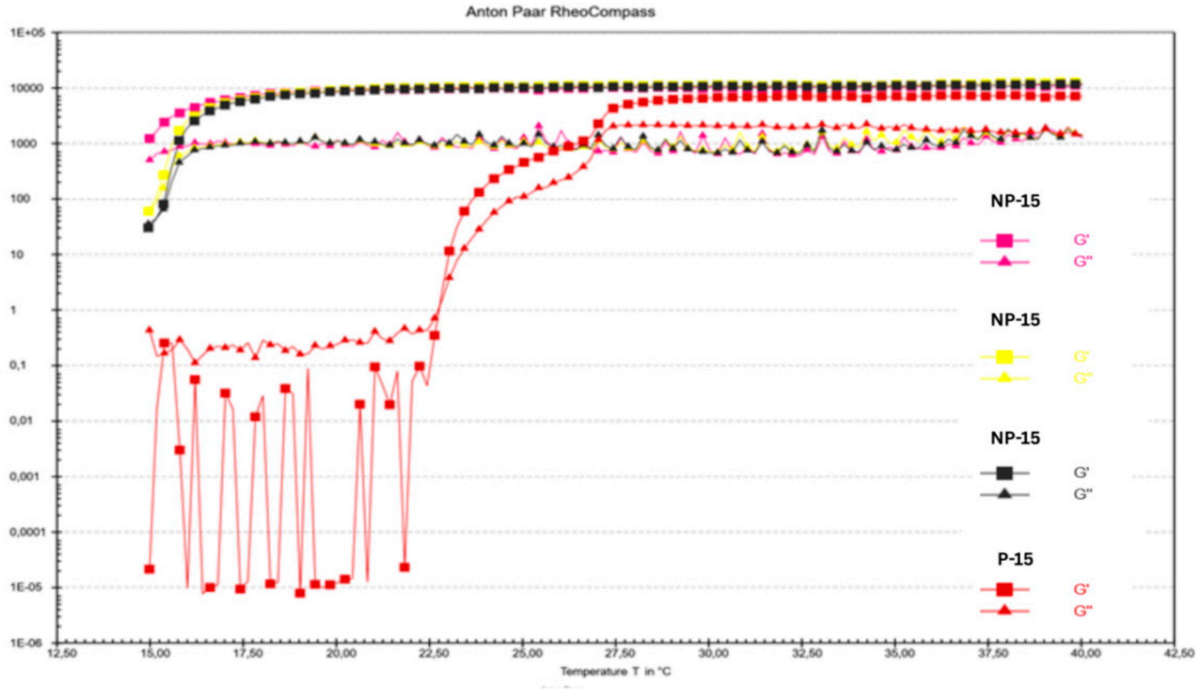

Figure S2 (a). Temperature sweep curves of 15% P407 loaded and blank gels

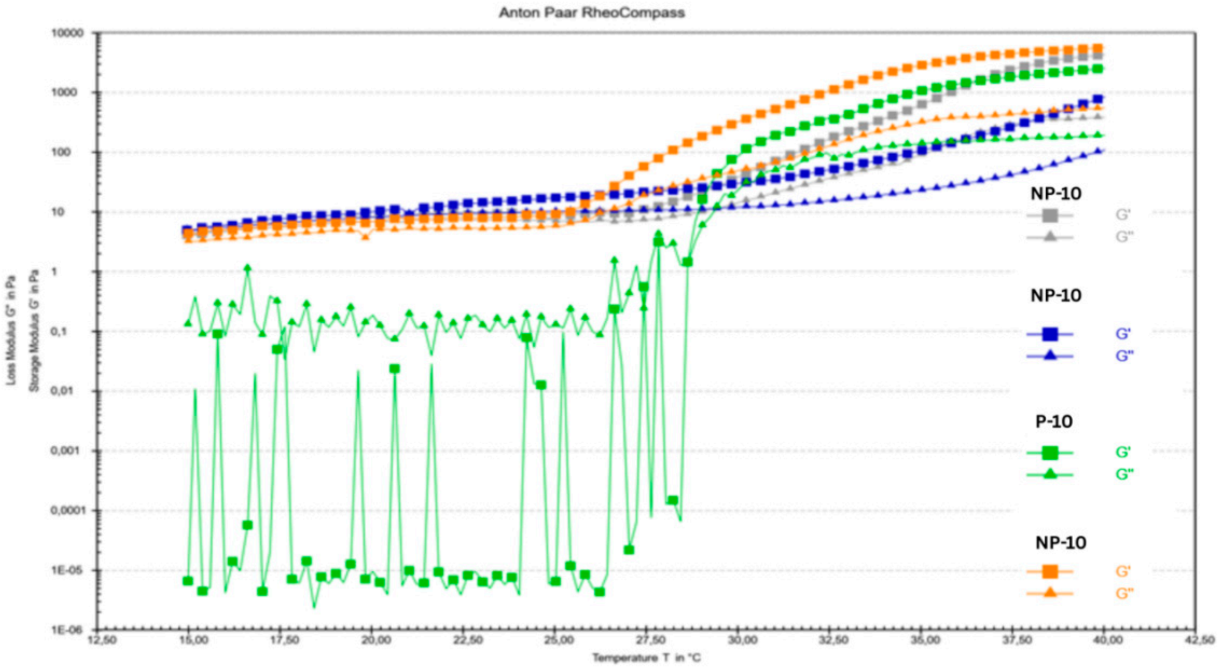

Figure S2 (b). Temperature sweep curves of 10% P407 loaded and blank gels

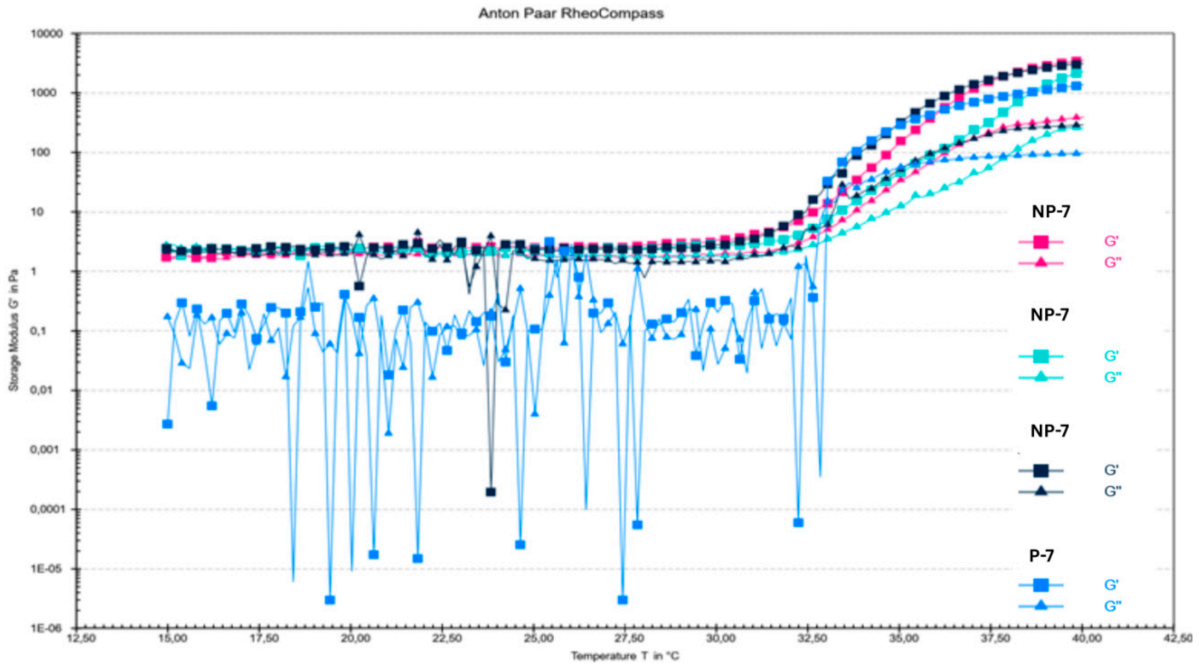

Figure S2 (c). Temperature sweep curves of 7% P407 loaded and blank gels

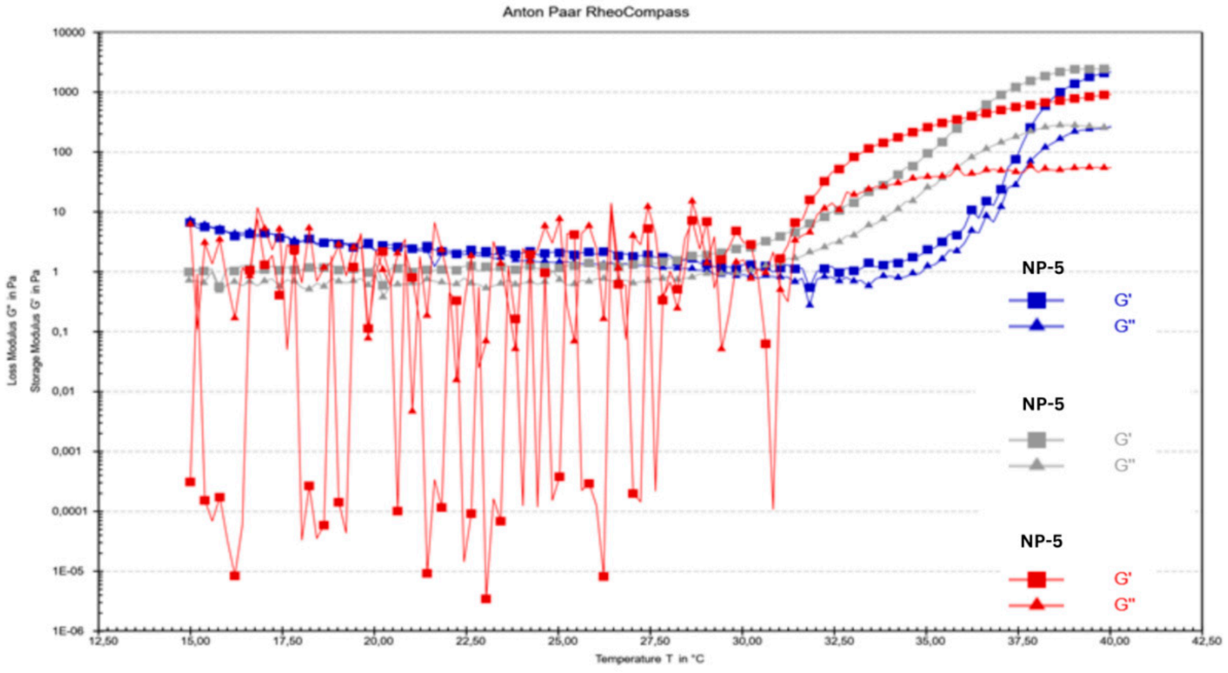

Figure S2 (d). Temperature sweep curves of 5% P407 loaded and blank gels

Time–Sweep Study

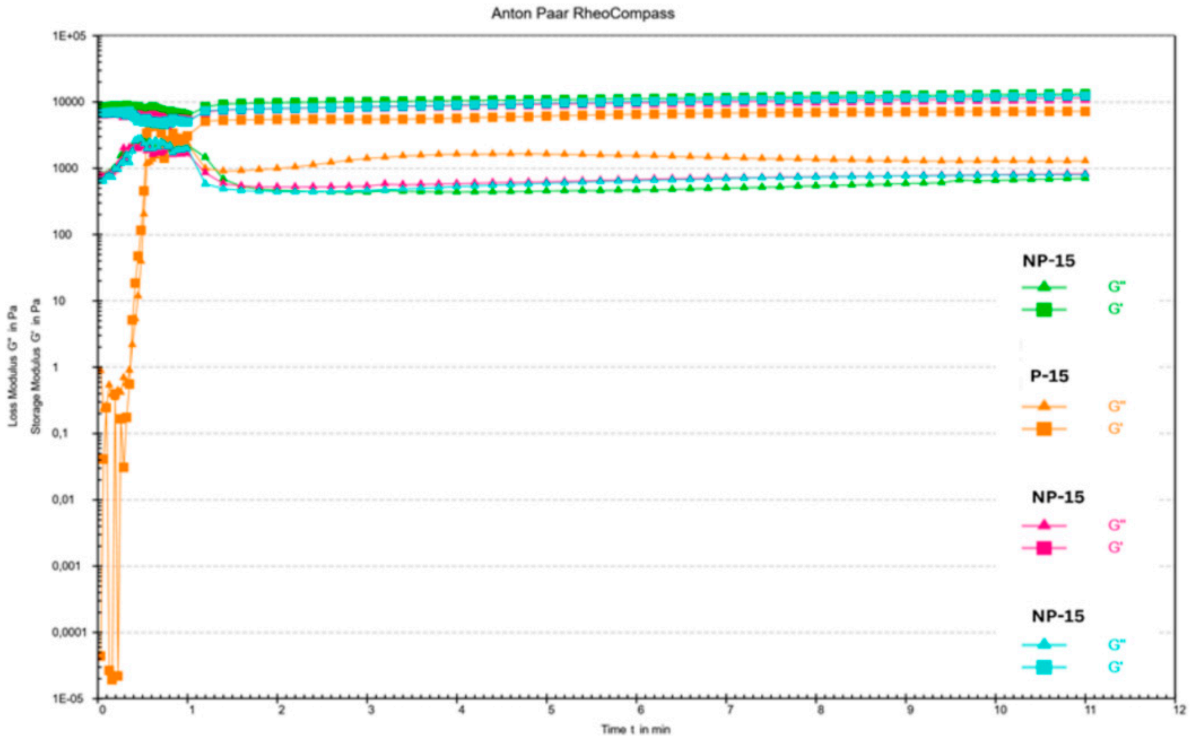

Figure S2 (e). Time sweep curves of 15% P407 loaded and blank gels

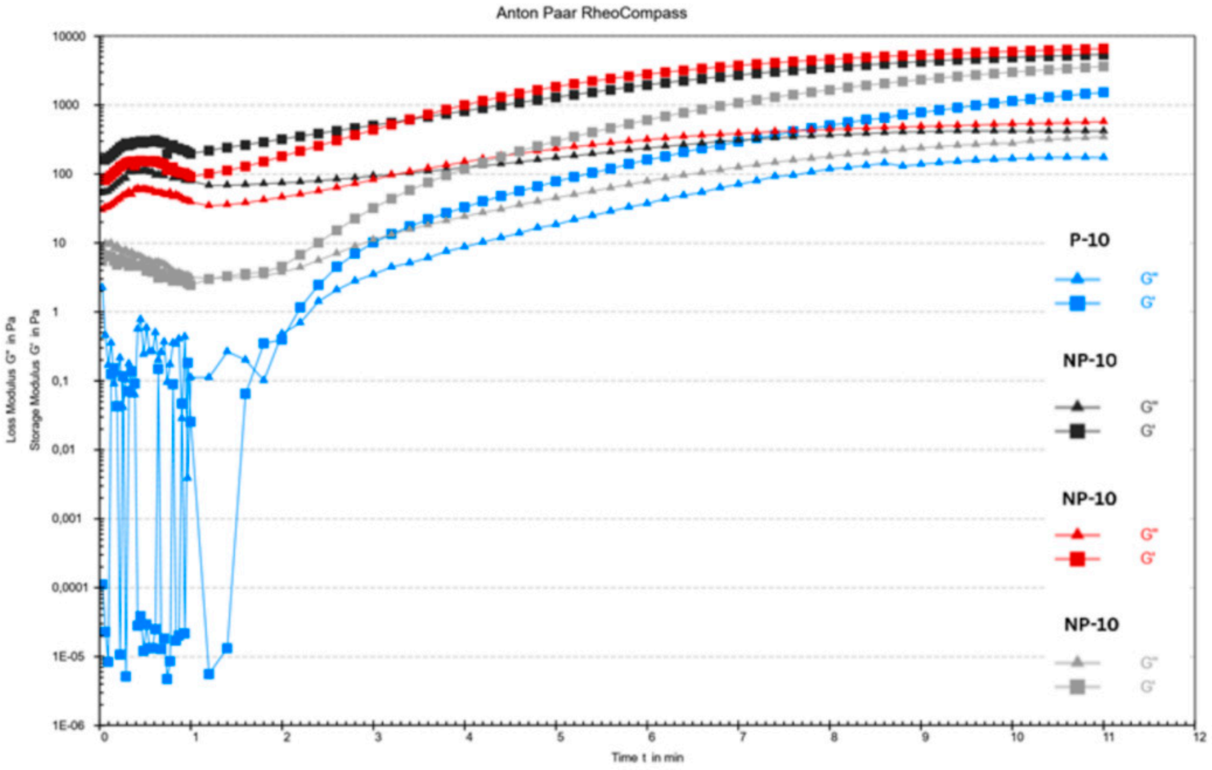

Figure S2 (f). Time sweep curves of 10% P407 loaded and blank gels

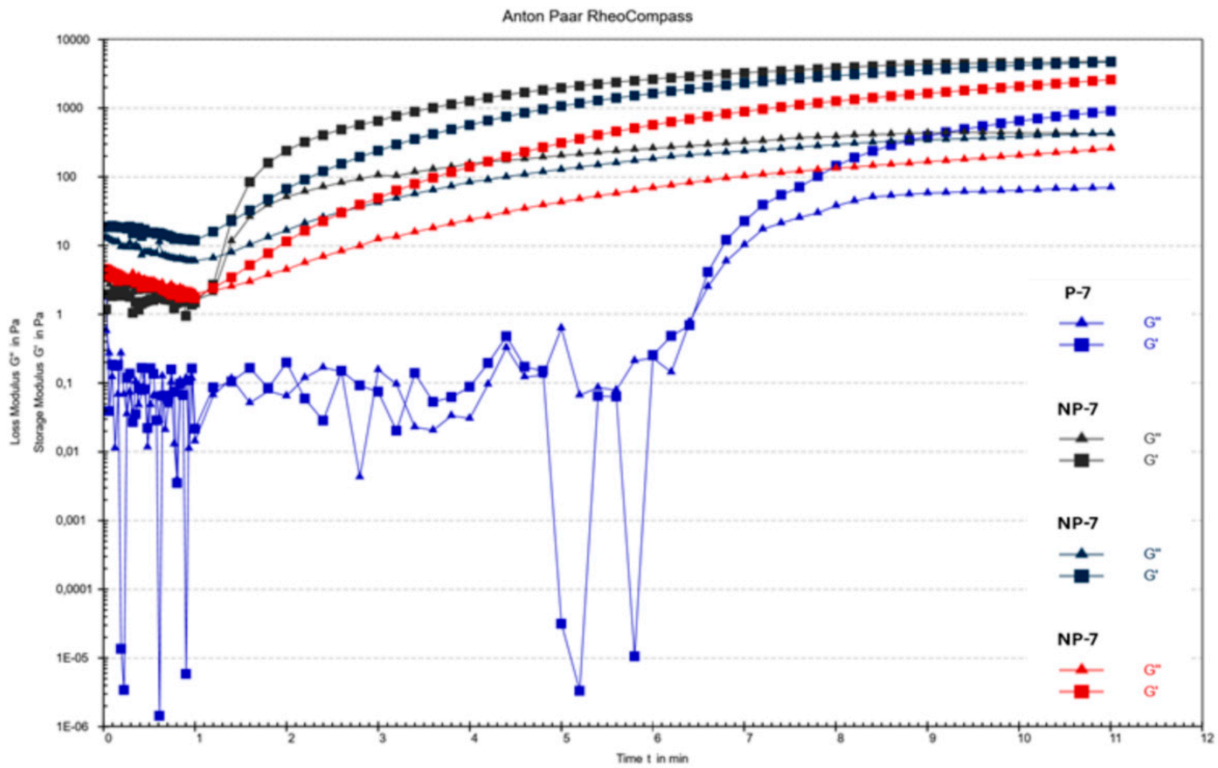

Figure S2 (g). Time sweep curves of 7% P407 loaded and blank gels

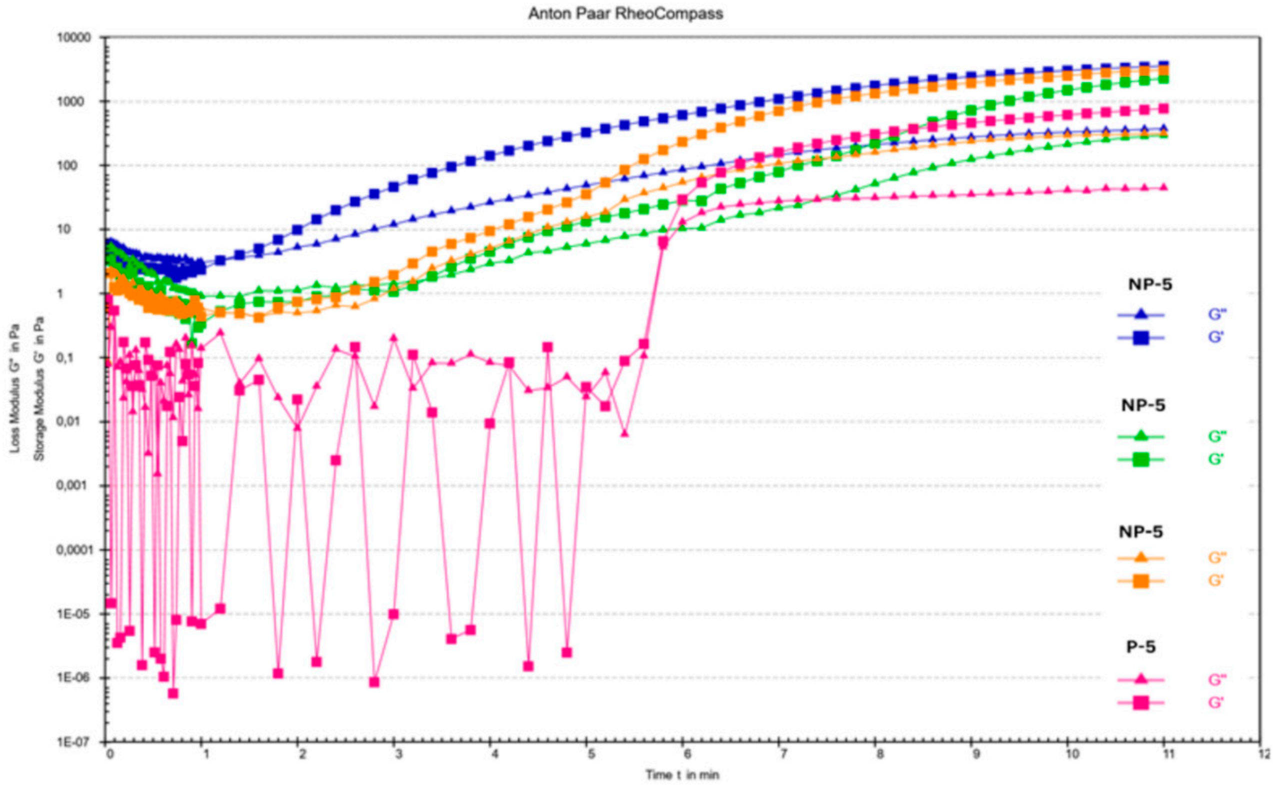

Figure S2 (h). Time sweep curves of 5% P407 loaded and blank gels

Figure S3. Antibacterial study results against three bacterial strains on agar media.

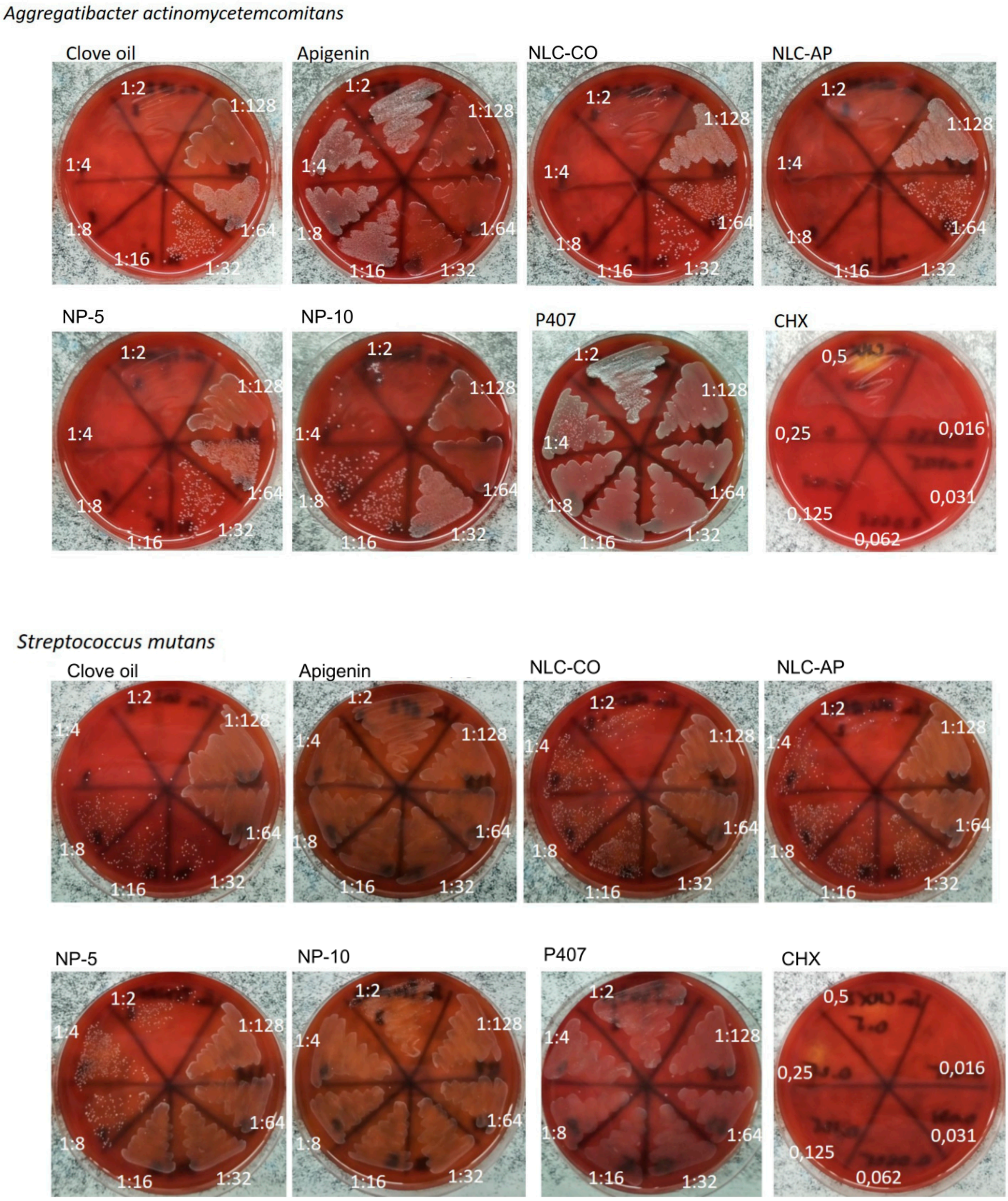

Eikenella corrodens

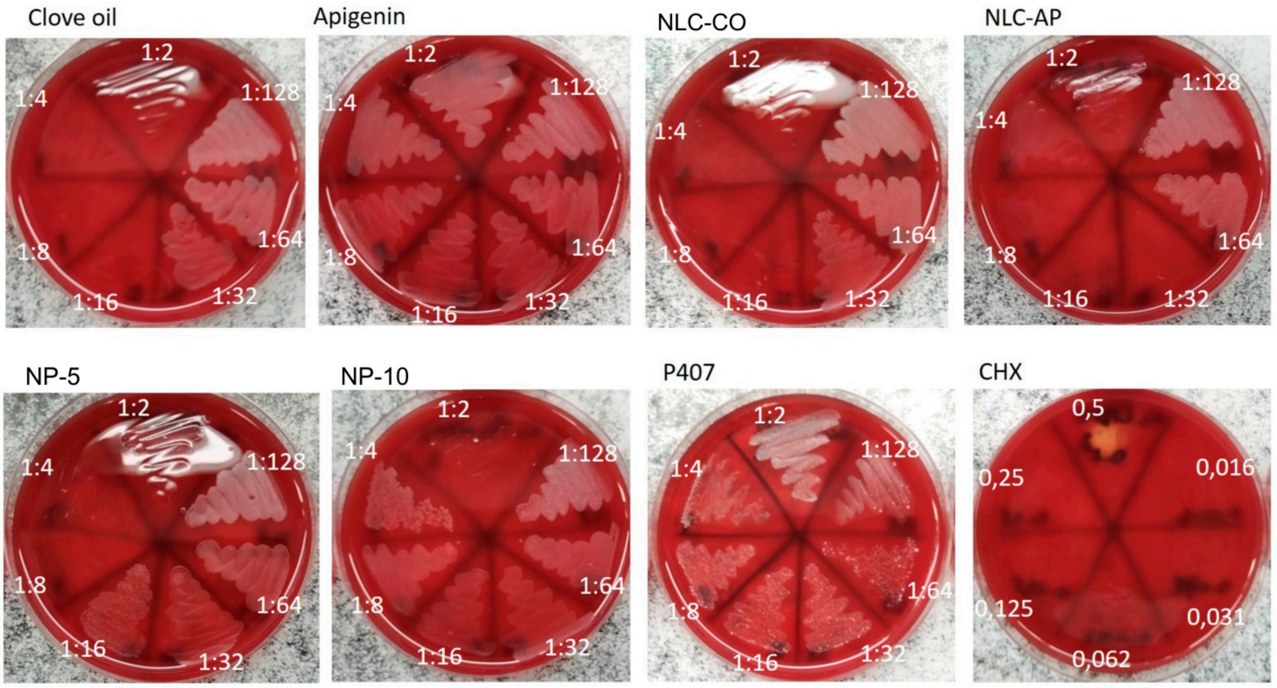

Supplement: Supplementary file 1 [file gels-12-00268-s001.zip › gels-4209082-supplementary.pdf]
